# Supplementary material for: Selection and structural characterization of anti-TREM2 scFvs that reduce levels of shed ectodomain
Source: Structure. 2021 Nov 4;29(11):1241–1252.e5. doi: 10.1016/j.str.2021.06.010 (PMC8575122; doi:10.1016/j.str.2021.06.010)
Supplement: Document S1. Figures S1–S6 and Table S1 [file mmc1.pdf]

Structure, Volume *29*

Supplemental Information

Selection and structural characterization of anti-TREM2 scFvs that reduce levels of shed ectodomain

Aleksandra Szykowska, Yu Chen, Thomas B. Smith, Charlotta Preger, Jingjing Yang, Dongming Qian, Shubhashish M. Mukhopadhyay, Edvard Wigren, Stephen J. Neame, Susanne Gräslund, Helena Persson, Peter J. Atkinson, Elena Di Daniel, Emma Mead, John Wang, John B. Davis, Nicola A. Burgess-Brown, and Alex N. Bullock

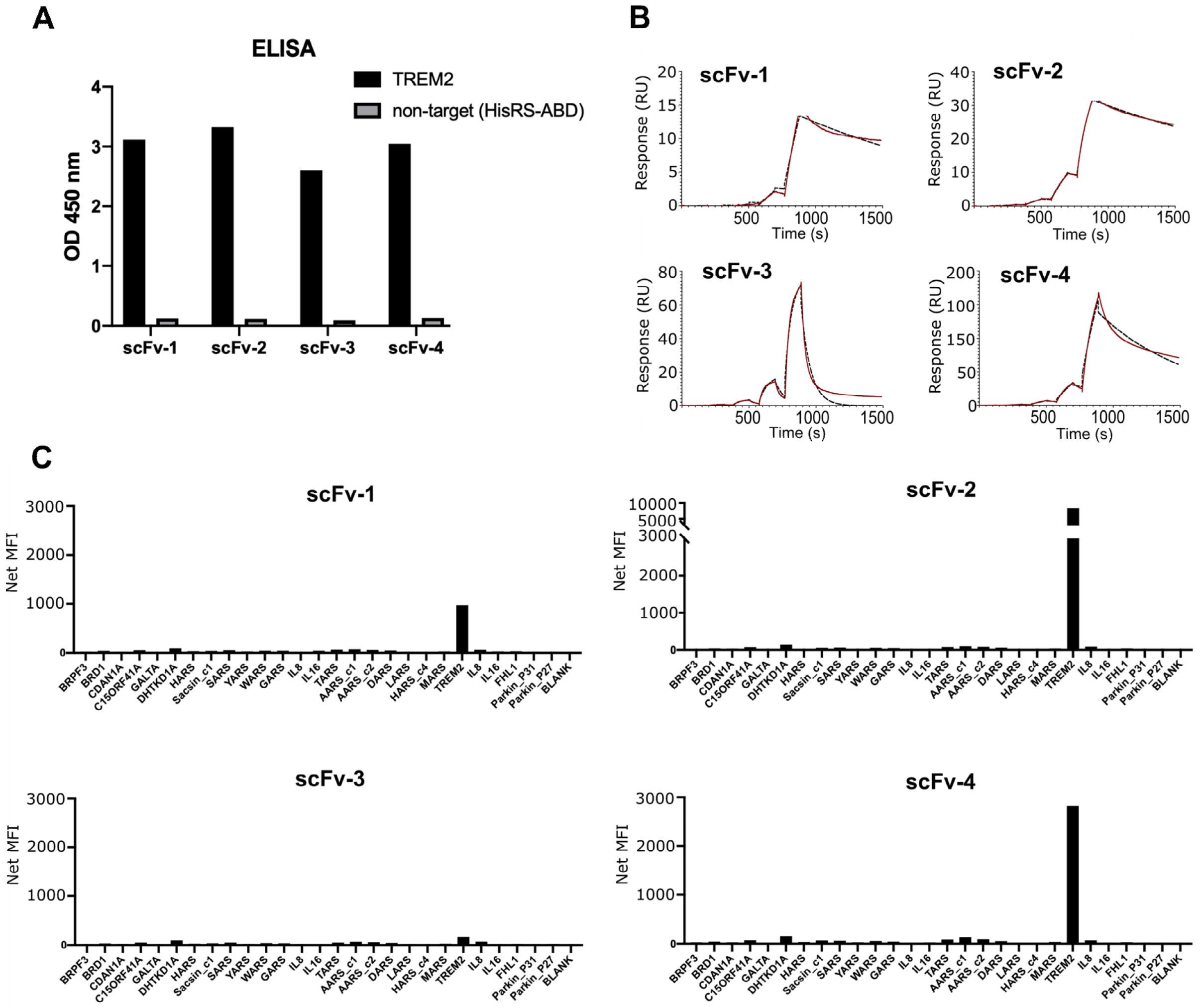

Figure S1. Initial validation of scFvs against TREM2, Related to STAR Methods. (A) Validation of scFvs binding to TREM2 by ELISA. Antigen TREM2 (a.a. 19-131) purified from insect cells (black bars) or a non-target protein (HisRS-ABD, grey bars) was added to a streptavidin-coated plate, followed by the addition of scFvs (in bacteria supernatant). Bound scFv were detected using anti-FLAG M2 HRP-conjugated antibody and TMB substrate. (**B**) Initial SPR Single Cycle Kinetic measurements of scFvs purified from *E. coli* using Biacore T200 (Cytiva). An anti-FLAG M2 antibody was immobilised on a CM5 sensor chip and FLAG-tagged scFv was captured on the surface. The same TREM2 antigen was injected with increasing concentrations (0.16, 0.8, 4, 20 and 100 nM*)*. Response was measured in resonance units (RU) over time (s). Red lines represent the measured data values, and dashed black lines represent the fit of the curve based on a 1:1 Langmuir binding model. (**C**) Luminex binding of scFvs against a panel of 27 proteins including TREM2. Proteins were added to neutravidin coupled magnetic colour-coded beads before addition of scFv (bacteria supernatant). Binding was detected using an RPE-conjugated anti-FLAG M2 antibody and analysed using FLEXMAP 3D (Luminex corp.) recording median fluorescence intensity (MFI).

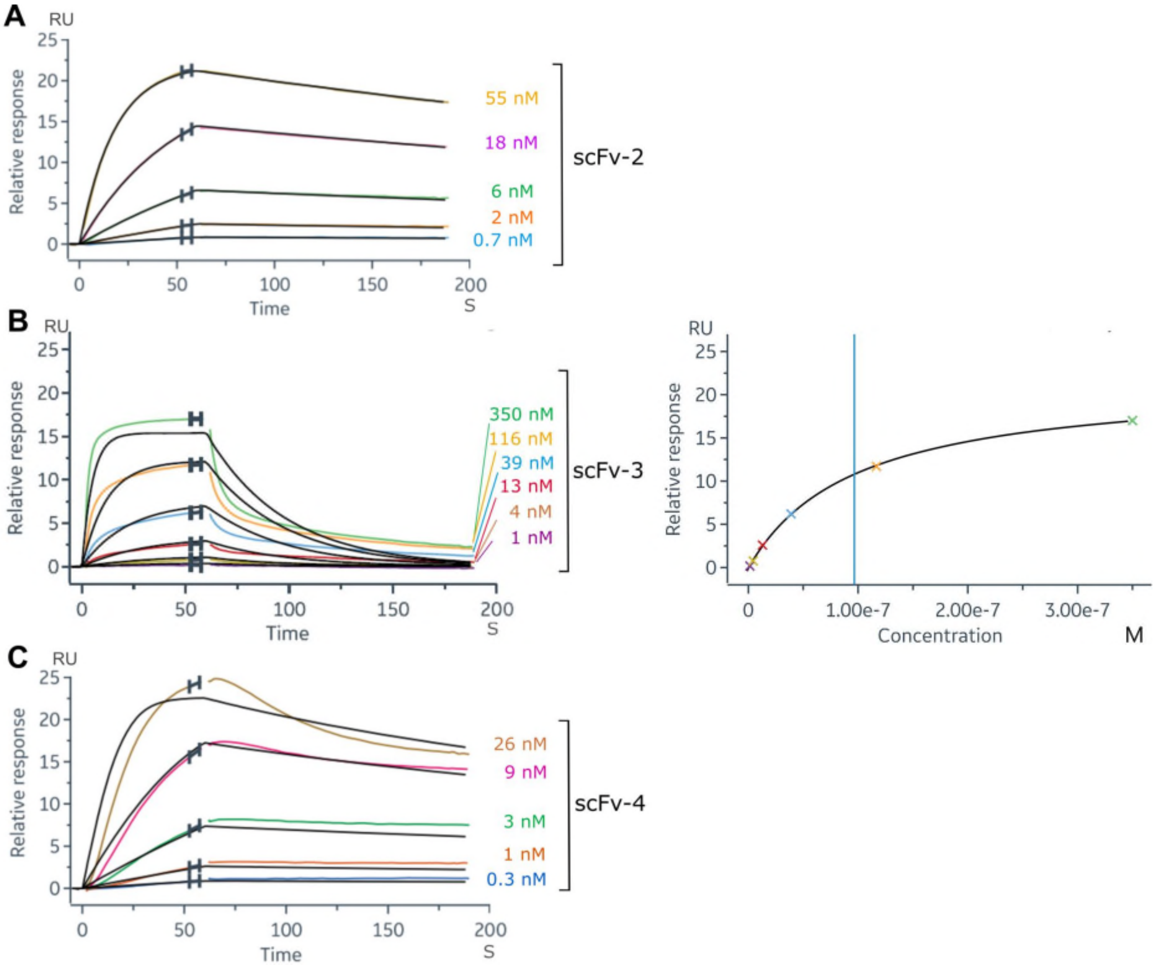

Figure S2. Kinetic measurements of three anti-TREM2 scFvs and estimation of apparent K_D values, Related to STAR Methods. Binding kinetics were determined by surface plasmon resonance (SPR) performed on a Biacore 8K instrument. ScFvs were produced in insect cells and showed a mix of monomeric and dimeric species. Biotinylated TREM2 (His19-Ser174) produced in Expi293F™ (Thermofisher Scientific, A14527) was immobilised using the Biotin CAPture Kit (Cytiva) and the binding of different concentrations of scFv was analysed. The binding kinetics of (**A**) scFv-2, (**B**) scFv-3 and (**C**) scFv-4 to TREM2 were fitted using a Langmuirian 1:1 model. ScFv-2: k_{on} = 9.9 x 10^5 $M^{-1}s^{-1}$, k_{off} = 1.6 x 10^{-3} s^{-1}, $^{App}K_D$ = 1.6 nM; ScFv-3: k_{on} = 5.0 x 10^5 $M^{-1}s^{-1}$, k_{off} = 2.7 x 10^{-2} s^{-1}, $^{App}K_D$ = 54 nM; ScFv-4: k_{on} = 5.0 x 10^6 $M^{-1}s^{-1}$, k_{off} = 2.9 x 10^{-3} s^{-1}, $^{App}K_D$ = 0.6 nM. The data for scFv-3 fit relatively poorly to a 1:1 model. However, steady state equilibrium analysis (right panel) yielded similar $^{App}K_D$ = 96 nM. Some non-specific interaction to the reference chip was observed for both scFv-3 and scFv-4. The k_{on} and k_{off} may also contain an avidity component, especially in the case of scFv-3 and scFv-4, so $^{App}K_D$ is used to describe the results. Association and dissociation curves are coloured for each concentration and the fitted curves are drawn in black. Competitive binding studies suggested that scFv-3 and scFv-4 compete for binding to TREM2 (data not shown).

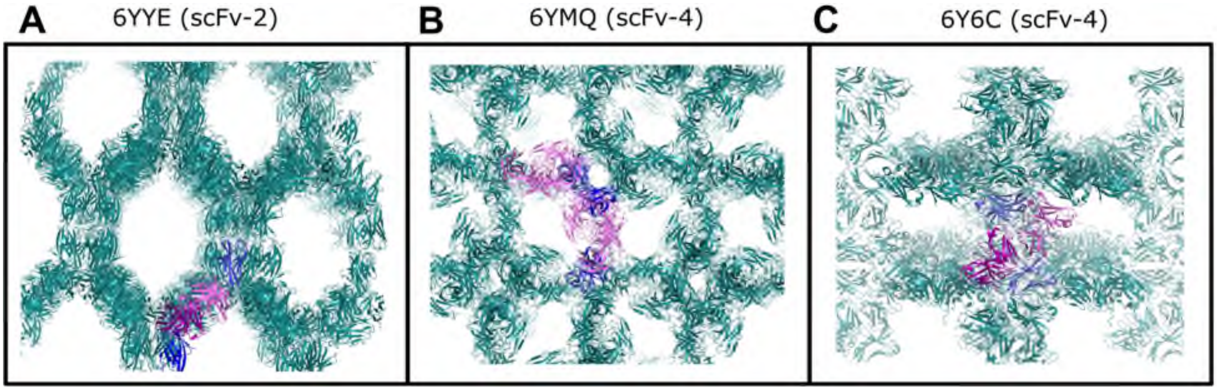

Figure S3. The crystal lattices of scFvs and TREM2 complexes, Related to Figure 3. (**A**) Crystal lattice of scFv-2 and TREM2 complex (6YYE) crystallised in unit cell of 114, 126, 225 Å with 77% solvent composition. Lattice was visualised by generating symmetry mates of components of an asymmetric unit consisting of 2 subunits of scFv (pink) and six subunits of TREM2 (blue). (**B**) Crystal lattice of the scFv-4 and TREM2 complex (6YMQ) crystallised in unit cell of 167, 181, 125 Å with 69% solvent composition. Green lattice visualised by generating symmetry mates of components of an asymmetric unit consisting of 6 subunits of scFv (pink) and six subunits of TREM2 (blue) (**C**) Crystal lattice of scFv-4 and TREM2 complex (6Y6C) crystallised in unit cell of 112, 112, 232 Å with 74% solvent composition. Lattice was visualised by generating symmetry mates of components of an asymmetric unit consisting of 2 subunits of scFv (pink) and two subunits of TREM2 (blue).

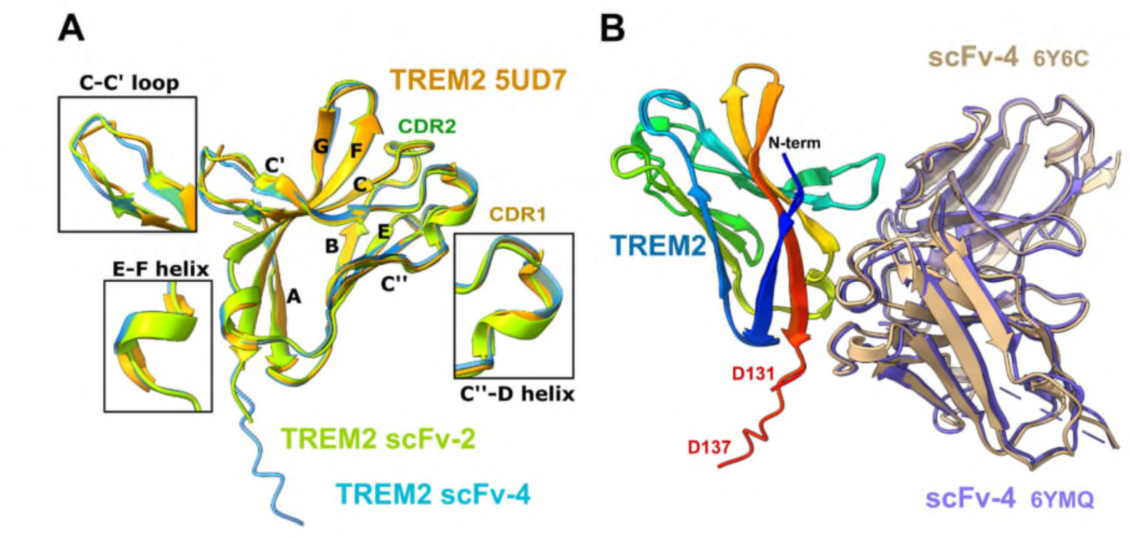

Figure S4. Superimposition of selected TREM2 and scFv-2 and scFv-4 co-structures, Related to Figure 3. **(A)** Superimposition of the highest resolution structure of TREM2 (2.2 Å) previously deposited in the PDB, 5UD7 (Sudom *et al.*, 2018), and TREM2 bound to scFv-2 and scFv-4. The root mean square deviation (RMSD) of C-alpha atoms comparing 5UD7 to the TREM2 complexes with scFv-2 and scFv-4 is 0.7 Å and 0.4 Å, respectively. The loops displaying the highest variation are shown in inset panels and named by secondary structure or TREM2 CDR. Amongst them is the C-C’ loop which is differentially bound by the scFvs as well as the CDR2 loop which shows a variable extent of a short α-helix, especially when bound to scFv-2. TREM2 5UD7 is coloured in orange, scFv-4-bound TREM2 in blue and scFv-2-bound TREM2 in green. **(B)** Superposition of the two TREM2-scFv-4 structures showed excellent agreement (Cα RMSD = 0.5 Å). ScFv-4 in complex with TREM2 His19-Ser174 (6Y6C) is shown in tan, whereas scFv-4 in complex with TREM2 His19-Asp131 (6YQM) is shown in purple.

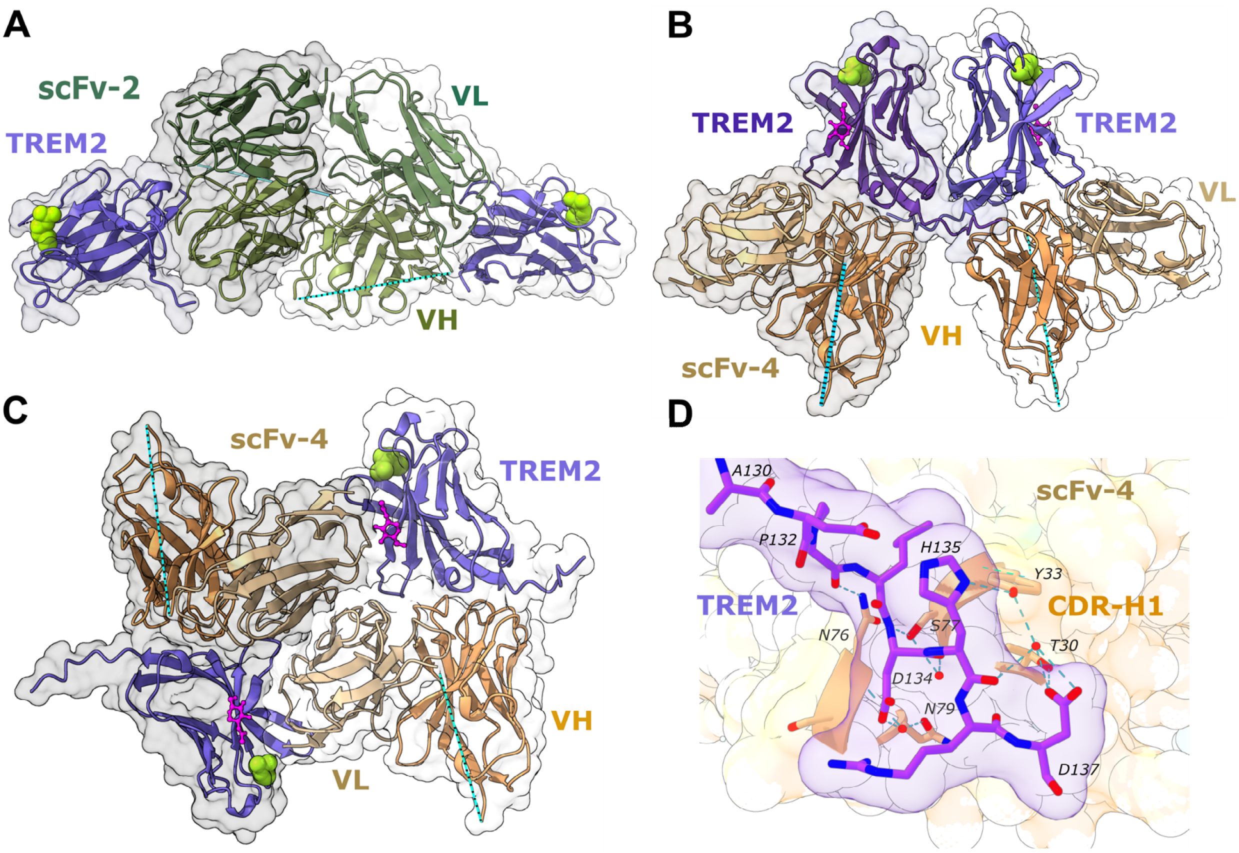

Figure S5. Different crystal packing of TREM2-scFv2 and scFv-4 complexes, Related to Figure 3. (**A**) Crystal packing between scFv-2 subunits in the TREM2 complex (PDB 6YYE). The scFv-2 VH and VL domains are coloured in different shades of green; The disordered linker between VH and VL domains is shown by a dotted cyan line. TREM2 is coloured slate blue. A molecular surface representation of each TREM2-scFv-2 complex is shown in a different transparent shade of grey. The TREM2 side chain Arg47 is shown in green spacefill for reference. (**B**) Crystal packing between TREM2 (a.a. 19-174, slate blue) and scFv-4 subunits (tan)(PDB 6Y6C). Each TREM2 subunit interacts with two scFv-4 molecules through interactions from the TREM2 ectodomain and stalk region, respectively. N-acetylglucosamine (NAG) is coloured in pink and presented as ball and stick (**C**) Crystal packing between scFv-4 subunits (tan) observed in the TREM2 (a.a. 19-174) co-structure (PDB 6Y6C). Each scFv-4 subunit packs against two molecules of TREM2 by interaction with the primary TREM2 epitope as well as an additional surface consisting of the TREM2 C-C' and F-G loops. (**D**) The TREM2 C-terminal stalk sequence Pro132-Asp137 (purple) packs against ScFv-4 CDR-H1 and Asn76-Asn79 (tan). Residues forming hydrogen bond interactions are presented as sticks and waters as red spheres. Hydrogen bonds are shown as blue dashed lines.

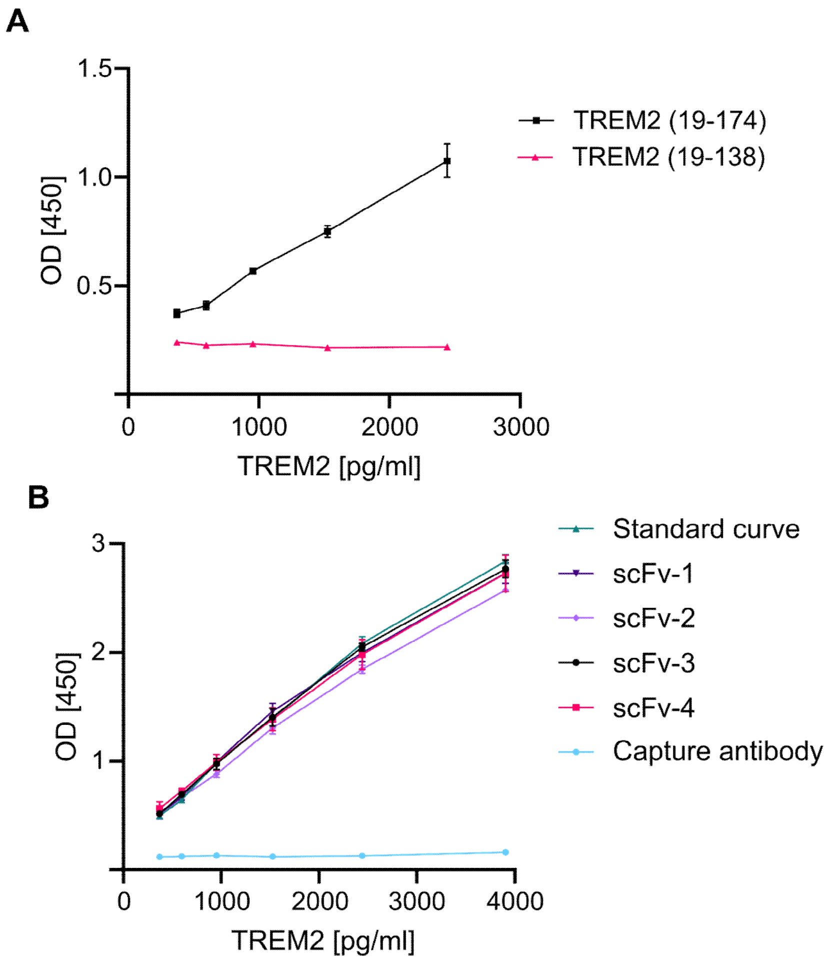

Figure S6. Validation of the sTREM2 ELISA assay, Related to Figure 6. (**A**) Identification of the soluble TREM2 amino acid sequence essential for ELISA detection. ELISA was performed using TREM2 (a.a. 19-174) protein containing the immunoglobulin domain and stalk and a shorter Ig domain only TREM2 protein (a.a. 19-138) in order to determine the requirement for the stalk sequence. The data represent n=1, 2 technical repeats. (**B**) Characterisation of epitope compatibility between ELISA antibodies and scFvs. Each of the four scFvs was incubated at 10 µg/mL with 3906 pg/mL standard curve peptide for 15 min. Dilution series were used to generate curves and a standard curve containing TREM2 peptide only was used for comparison. The capture antibody added to the standard curve is used to demonstrate the effects of overlapping epitopes. The data represent n=1, 2 technical repeats, except for scFv-3 and scFv-4 where n=2 independent experiments.

Table S1. Oligonucleotide sequences used for cloning, related to Key Resources Table

OLIGONUCLEOTIDE	SOURCE	IDENTIFIER
scFvs scaffold-fwd	SourceBioscience	TACTTCCAATCCATGGAGGTGCAATTGTTGGAGAGC
scFvs scaffold-rvs	SourceBioscience	TATCCACCTTTACTGTCATTTGATCTCCAGCTTGGT
pHL-sec-signal peptide forward	SourceBioscience	ATGGGGATCCTTCCCAGCCCTGGGATGCCTGCGCTGCTCTCCCTCGTGAGCCTTCTCT
pHL-sec-signal peptide reverse	SourceBioscience	ACCGGTTTCAGCTACGCAACCCATCAGCAGCACGGAGAGAAGGCTCACGAGGGAGAG
pTT5-TREM2 (19-131) forward	SourceBioscience	GATGGGTTGCGTAGCTGAAACCGGTCACAACACCACCGTGTTCCAGGGCGT
pTT5-TREM2 (19-131) reverse	SourceBioscience	CTCGAGTGATCATTAGTGATGGTGATGGTGGTGCTTGGTACCGTCTGCCAGCACCTCCACCAGGAC
pTT5-TREM2 (1-174) forward	SourceBioscience	ACGGATCTCTAGCGAATTCACCATGGAGCCTCTGCGCCTGCTGATC
pTT5-TREM2(1-174) reverse	SourceBioscience	CAATTTTCTGAGCTTCGAAGATGTCGTTCAGACCGGAAGTCGGTGGGAACGGGATTTCAC
pHTBV1.1sec-TREM2 (19-174) reverse	SourceBioscience	TATCCACCTTTACTGCTGGAAGTGGGTGGGAAGGG
pHTBV1.1sec-TREM2 (19-174) forward	SourceBioscience	TACTTCCAATCCATGCACAACACCACAGTGTTCC
pFB-sec-bio-TREM2 (1-131) forward	SourceBioscience	As above
pFB-sec-bio-TREM2 (1-138) forward	SourceBioscience	As above
pFB-sec-bio-TREM2 (1-131) reverse	SourceBioscience	TATCCACCTTTACTGCTGTCTGCCAGCACCTCCAC
pFB-sec-bio-TREM2 (1-138) reverse	SourceBioscience	TATCCACCTTTACTGCTAGCATCCCGGTGATCCAG
pFB-sec-NH-TREM2 (1-174) forward	SourceBioscience	TTAAGAAGGAGATATACTATGGAGCCTCTCCGGCTGCTC
pFB-sec-NH-TREM2 (1-174) reverse	SourceBioscience	GATTGGAAGTAGAGGTTCTCTGCCCCGGGGAACCAGAGATC
