# Supplementary material for: Selection and structural characterization of anti-TREM2 scFvs that reduce levels of shed ectodomain
Source: Structure. 2021 Nov 4;29(11):1241–1252.e5. doi: 10.1016/j.str.2021.06.010 (PMC8575122; doi:10.1016/j.str.2021.06.010)
Supplement: Document S2. Article plus supplemental information [file mmc2.pdf]

Structure

Article

Selection and structural characterization of anti-TREM2 scFvs that reduce levels of shed ectodomain

Graphical abstract

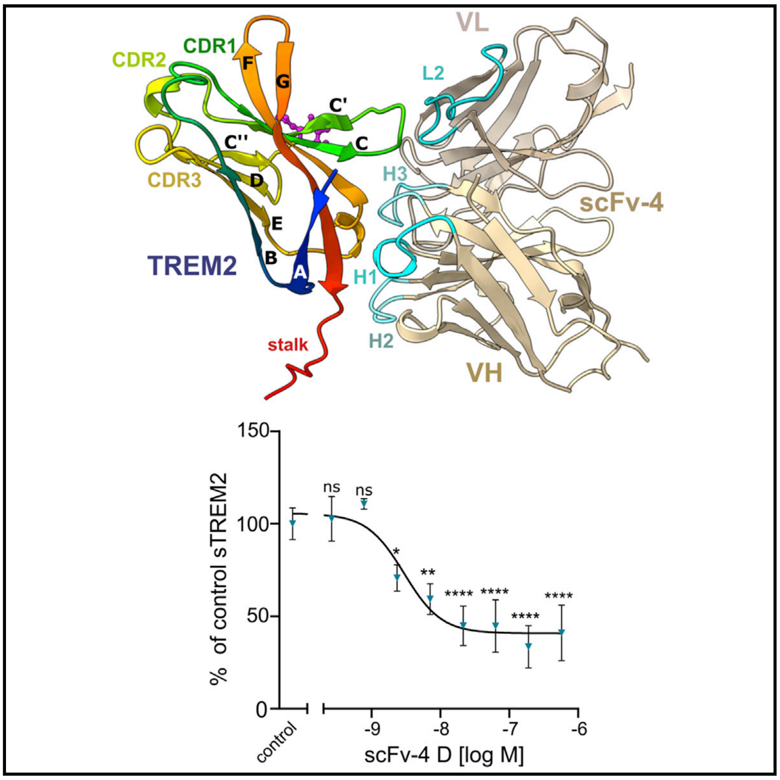

Authors

Aleksandra Szykowska, Yu Chen, Thomas B. Smith, ..., John B. Davis, Nicola A. Burgess-Brown, Alex N. Bullock

Correspondence

john.davis@cmd.ox.ac.uk (J.B.D.),
nicola.burgess-brown@cmd.ox.ac.uk (N.A.B.-B.),
alex.bullock@cmd.ox.ac.uk (A.N.B.)

In brief

TREM2 variants are associated with an increased risk of dementia. Szykowska et al. generated scFv antibody fragments to TREM2 immunoglobulin-like domain and solved crystal structures of scFv-TREM2 complexes to reveal their binding modes. Oligomeric scFv species showed enhanced functional activity reducing levels of shed TREM2 ectodomain in a HEK293 model.

Highlights

- scFvs were generated against the immunoglobulin-like domain of the receptor TREM2
- Crystal structures revealed scFv binding to epitopes outside the TREM2 CDRs
- Oligomeric scFv species reduced levels of shed TREM2 ectodomain in a HEK293 model
- The scFvs form renewable structural and functional biology tools for TREM2 research

Szykowska et al., 2021, Structure *29*, 1241–1252
November 4, 2021 © 2021 The Authors. Published by Elsevier Ltd.
https://doi.org/10.1016/j.str.2021.06.010

Structure

CellPress
OPEN ACCESS

Article

Selection and structural characterization of anti-TREM2 scFvs that reduce levels of shed ectodomain

Aleksandra Szykowska,[1] Yu Chen,[2] Thomas B. Smith,[3,1] Charlotta Preger,[4] Jingjing Yang,[5] Dongming Qian,[5] Shubhashish M. Mukhopadhyay,[1] Edvard Wigren,[4] Stephen J. Neame,[6] Susanne Gräslund,[4] Helena Persson,[7] Peter J. Atkinson,[6] Elena Di Daniel,[3,1] Emma Mead,[3,1] John Wang,[2] John B. Davis,[3,1,*] Nicola A. Burgess-Brown,[1,*] and Alex N. Bullock[1,8,*]

[1]Centre for Medicines Discovery, Nuffield Department of Medicine, University of Oxford, Old Road Campus Research Building, Roosevelt Drive, Oxford OX3 7DQ, UK
[2]Eisai Inc., 35 Cambridgepark Drive, Cambridge, MA 02140, USA
[3]Alzheimer's Research UK Oxford Drug Discovery Institute, NDM Research Building, University of Oxford, Old Road Campus, Roosevelt Drive, Oxford OX3 7FZ, UK
[4]Structural Genomics Consortium (SGC), Karolinska Institutet, Karolinska University Hospital, Division of Rheumatology, Department of Medicine Solna, 171 76 Stockholm, Sweden
[5]Viva Biotech Ltd., 334 Aidisheng Road, Zhangjiang High-Tech Park, Shanghai 201203, China
[6]Eisai Ltd., Mosquito Way, Hatfield AL10 9SN, UK
[7]Science for Life Laboratory, Drug Discovery and Development & School of Engineering Sciences in Chemistry, Biotechnology and Health, Royal Institute of Technology (KTH), Stockholm, Sweden
[8]Lead contact
*Correspondence: john.davis@cmd.ox.ac.uk (J.B.D.), nicola.burgess-brown@cmd.ox.ac.uk (N.A.B.-B.), alex.bullock@cmd.ox.ac.uk (A.N.B.)
https://doi.org/10.1016/j.str.2021.06.010

SUMMARY

Mutations in TREM2, a receptor expressed by microglia in the brain, are associated with an increased risk of neurodegeneration, including Alzheimer's disease. Numerous studies support a role for TREM2 in sensing damaging stimuli and triggering signaling cascades necessary for neuroprotection. Despite its significant role, ligands and regulators of TREM2 activation, and the mechanisms governing TREM2-dependent responses and its cleavage from the membrane, remain poorly characterized. Here, we present phage display generated antibody single-chain variable fragments (scFvs) to human TREM2 immunoglobulin-like domain. Co-crystal structures revealed the binding of two scFvs to an epitope on the TREM2 domain distal to the putative ligand-binding site. Enhanced functional activity was observed for oligomeric scFv species, which inhibited the production of soluble TREM2 in a HEK293 cell model. We hope that detailed characterization of their epitopes and properties will facilitate the use of these renewable binders as structural and functional biology tools for TREM2 research.

INTRODUCTION

Late-onset Alzheimer's disease (LOAD) is the most common type of dementia, characterized by accumulation of extracellular amyloid-β (Aβ) aggregates and intracellular neurofibrillary tangles of hyper-phosphorylated tau, with a long prodromal phase followed by cognitive decline. Despite the urgency and necessity to develop therapeutics, there are currently no approved drugs which cure or slow the underlying progression of Alzheimer's disease (AD) and patients are still reliant on symptomatic treatments discovered in the mid-last century (Long and Holtzman, 2019).

After some late-stage clinical trials targeting Aβ failed to meet their desired endpoints and several large genome-wide association studies (GWAS) linked genes coding for components of the immune response to AD, neuro-inflammation has become an area of intense research for therapeutics (Block et al., 2007; Lambert et al., 2013). Among the genes identified by GWAS is *TREM2* (Triggering Receptor Expressed on Myeloid cells), which encodes a single transmembrane receptor expressed in myeloid-derived cells, including microglia in the central nervous system (CNS) (Guerreiro et al., 2013). Homozygous loss-of-function mutations in TREM2, or the associated adaptor protein DAP12, were previously identified to cause aggressive early-onset dementia in Nasu-Hakola disease (Paloneva et al., 2003). Since then, several point mutations in the extracellular domain of TREM2 have been linked to neurodegenerative disorders, highlighting the importance of TREM2 functions in brain health and homeostasis (Konishi and Kiyama, 2018; Ulrich and Holtzman, 2017). The most notable amino acid substitution, R47H, leads to

This is an open access article under the CC BY license (http://creativecommons.org/licenses/by/4.0/).

```
                                        CDR-H1                            CDR-H2
scFv-1   EVQLLESGGGLVQPGGSLRLSCAASGFTFSSYGMSWVRQAPGKGLEWVSGISSYGSYTYY   60
scFv-2   EVQLLESGGGLVQPGGSLRLSCAASGFTFYSSYMGWVRQAPGKGLEWVSYISSSGSSTYY   60
scFv-3   EVQLLESGGGLVQPGGSLRLSCAASGFTFPYYYMSWVRQAPGKGLEWVSSIYGSGGGTYY   60
scFv-4   EVQLLESGGGLVQPGGSLRLSCAASGFTFSYYYMGWVRQAPGKGLEWVSGISPSSGYTYY   60
         *****************************    *.************** *   .. ***

                                                    CDR-H3
scFv-1   ADSVKGRFTISRDNSKNTLYLQMNSLRAEDTAVYYCARHGSGGWDGYYFDYWGQGTLVTV   120
scFv-2   ADSVKGRFTISRDNSKNTLYLQMNSLRAEDTAVYYCARVGGYYSWGNGIDYWGQGTLVTV   120
scFv-3   ADSVKGRFTISRDNSKNTLYLQMNSLRAEDTAVYYCARGGLG--YGPYFDYWGQGTLVTV   118
scFv-4   ADSVKGRFTISRDNSKNTLYLQMNSLRAEDTAVYYCARYYYGYYY-SHMDYWGQGTLVTV   119
         **************************************          :***********

            LINKER                                    CDR-L1
scFv-1   SSGGGGSGGGGSGGGGSDIQMTQSPSSLSASVGDRVTITCRASQSISSYLNWYQQKPGKA   180
scFv-2   SSGGGGSGGGGSGGGGSDIQMTQSPSSLSASVGDRVTITCRASQSISSYLNWYQQKPGKA   180
scFv-3   SSGGGGSGGGGSGGGGSDIQMTQSPSSLSASVGDRVTITCRASQSISSYLNWYQQKPGKA   178
scFv-4   SSGGGGSGGGGSGGGGSDIQMTQSPSSLSASVGDRVTITCRASQSISSYLNWYQQKPGKA   179
         ************************************************************

            CDR-L2                                       CDR-L3
scFv-1   PKLLIYAASSLQSGVPSRFSGSGSGTDFTLTISSLQPEDFATYYCQQY--YYPPTFGQGT   238
scFv-2   PKLLIYAASSLQSGVPSRFSGSGSGTDFTLTISSLQPEDFATYYCQQYGVYYPFTFGQGT   240
scFv-3   PKLLIYAASSLQSGVPSRFSGSGSGTDFTLTISSLQPEDFATYYCQQT--YSLHTFGQGT   236
scFv-4   PKLLIYAASSLQSGVPSRFSGSGSGTDFTLTISSLQPEDFATYYCQQSR-SGLHTFGQGT   238
         ***********************************************       ******

scFv-1   KLEIK  243
scFv-2   KLEIK  245
scFv-3   KLEIK  241
scFv-4   KLEIK  243
         *****
```

Figure 1. Multiple sequence alignment of anti-TREM2 scFv protein sequences

ScFv sequences are aligned using Clustal O with the three heavy chains CDR-H1-3 highlighted in green and light chains CDRL1-3 in blue, and the 15 amino acid linker in gray. Boundaries for CDRs are as defined by the IMGT nomenclature (Lefranc et al., 2003). The scFvs originate from the human synthetic phage library SciLifeLib (Preger et al., 2020), in which sequence diversity was introduced into four of the six CDRs: CDR-H1, CDR-H2, CDR-H3, and CDR-L3.

4-fold increased risk of developing LOAD (Jonsson et al., 2013; Guerreiro et al., 2013). A stronger genetic effect is observed only in carriers of apolipoprotein E (APOE) ε4, a potential TREM2 ligand, which has been implicated in TREM2 pathology (Krasemann et al., 2017; Shi and Holtzman, 2018; Parhizkar et al., 2019).

TREM2 has numerous putative functions, including regulation of lipid and cholesterol metabolism, phagocytosis of myelin and Aβ, and generation of a microglial barrier around Aβ plaques (Jay et al., 2017; Nugent et al., 2020; Ulland and Colonna, 2018; Ulland et al., 2017; Yeh et al., 2016; Yuan et al., 2016). TREM2 has been reported to bind ligands as diverse as Aβ, lipids, myelin, and lipoproteins (Wang et al., 2015; Yeh et al., 2016; Zhao et al., 2018). Recent findings have indicated that TREM2-dependent signaling is essential for the transcriptional definition of disease-associated microglia, a phenotype that is believed to be neuroprotective as it upregulates genes involved in phagocytosis (e.g., Keren-Shaul et al., 2017).

TREM2 contains an immunoglobulin-like (Ig-like) domain followed by a flexible stalk region, a transmembrane domain, and a short cytoplasmic tail. The stalk region can be cleaved by ADAM10/17 proteases to generate a soluble TREM2 fragment (sTREM2), while the C-terminal intramembranous domain is further cleaved by gamma-secretase (Wunderlich et al., 2013). sTREM2 can be detected in cerebrospinal fluid (Wang et al., 2020) and is increased in patients with neuronal injury or CNS inflammatory diseases (Piccio et al., 2008; Rauchmann et al., 2019). In addition, sTREM2 was found to be increased in patients at early symptomatic stages of AD and correlated well with levels of phosphorylated tau in patients with tau pathology (Rauchmann et al., 2019; Suárez-Calvet et al., 2016). The importance of TREM2 cleavage in AD pathology is highlighted by the H157Y polymorphism at the protease cleavage site, which leads to excessive shedding of sTREM2 and increased risk of AD (Schlepckow et al., 2017; Thornton et al., 2017). It is unclear whether the increased risk is due to the resulting increased generation of additional sTREM2, which might be a biologically active molecule (Zhong et al., 2019), alterations in intracellular signaling or functional properties of the remaining C-terminal fragments.

There is minimal published structural data for TREM2 ectodomain motifs responsible for ligand engagement or regulation of TREM2 functions (Kober et al., 2016; Sudom et al., 2018). While

A

	Yield (mg/L)	
	E.coli	Baculovirus
ScFv-1	0.9	16
ScFv-2	0.5	18
ScFv-3	1.4	15
ScFv-4	1	13

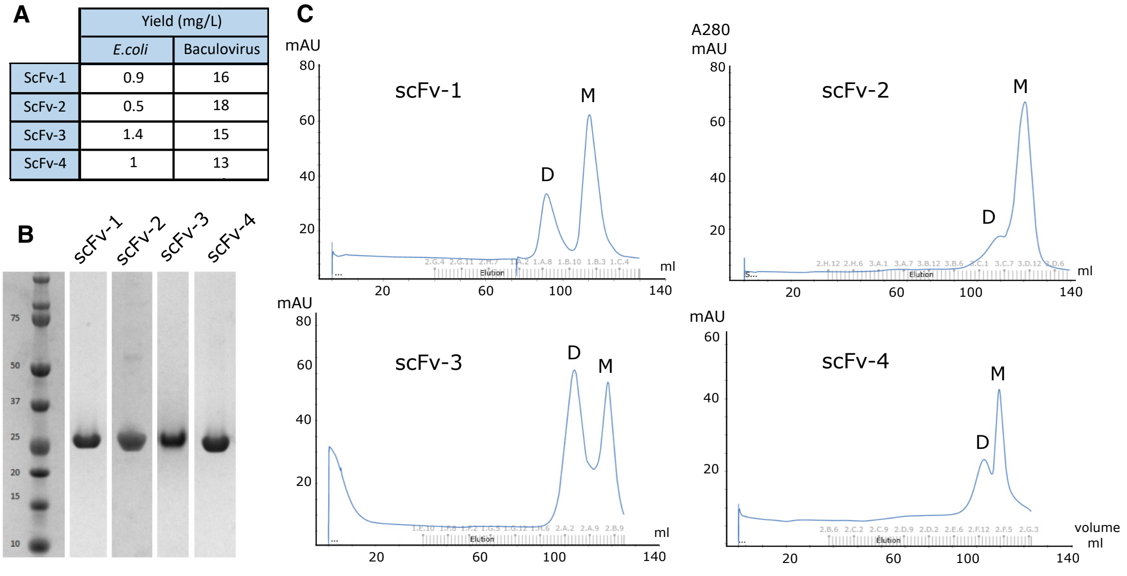

Figure 2. Optimization of scFv production in a baculoviral expression system

(A) Expression yields of scFvs produced in *E. coli* TOP10 cells or Sf9 insect cells.

(B) Coomassie blue-stained SDS-PAGE gel showing the final purity of scFvs after purification from Sf9 cells (image generated from different purification experiments).

(C) Size-exclusion chromatography of each scFv antibody fragment purified on a Superdex 200 16/60 column. Delayed elutions indicated scFv interactions with the Superdex media as well as a tendency for dimerization (D-dimer, M-monomer).

the Ig-like domain R47H mutant has been proposed to be defective in ligand binding, its crystal structure was solved only recently (Song et al., 2017; Sudom et al., 2018). The authors concluded that the arginine substitution in CDR1 causes extensive remodeling in the neighboring CDR2 loop of TREM2 resulting in local structural disorder and the loss of electron density. The same loop has been identified to interact with putative ligands in wild-type TREM2 crystals soaked with phosphatidylserine (Sudom et al., 2018). However, structures incorporating other ligands or the molecular mechanisms of TREM2 signaling remain to be fully elucidated.

Because of the potential therapeutic impact of targeting TREM2, we decided to generate single-chain variable antibody fragments (scFvs) against the human TREM2 ectodomain with which to study TREM2 structure and function. Agonist antibodies have already been reported in recently published work (Ellwanger et al., 2021; Fassler et al., 2021; Ibach et al., 2021; Price et al., 2020; Schlepckow et al., 2020; Wang et al., 2020). The group of Schlepckow have shown that a full-length antibody specific to mouse TREM2 can decrease shedding and activate TREM2 signaling *in vitro*, and also lead to a significant reduction in amyloid plaques in 6-month-old amyloid-beta precursor protein knockin mice (Schlepckow et al., 2020). Antibodies might also be useful biochemical tools for studying the function of sTREM2 (Zhong et al., 2019).

There are currently no published structures of antibodies in complex with TREM2. Here, we present our work on the generation and characterization of four scFvs selected against the Ig-like domain (herein Ig domain) of human TREM2. The co-crystal structures of two of these antibody fragments, scFv-2 and scFv-4, have been solved in complex with TREM2, revealing interactions with epitopes distal to the putative ligand-binding site. Two antibody fragments, scFv-3 and scFv-4, reduced the shedding of sTREM2 from HEK293 cells.

Together, these data identify scFv antibody fragments as valuable reagents for cellular and structural studies of the TREM2 receptor and identify TREM2/ligand interfaces that may be used to modulate TREM2 function.

RESULTS

Phage display selection of anti-TREM2 scFvs

To facilitate the development of renewable antibody binders against TREM2, we performed phage display selections using the SciLifeLib synthetic library of human scFvs similarly constructed and designed as reported previously (Preger et al., 2020; Säll et al., 2016). A TREM2 antigen comprising the Ig domain of human TREM2 (His19-Asp131) was expressed recombinantly from Sf9 insect cells and biotinylated on a C-terminal Avi tag for immobilization on streptavidin-coated magnetic beads. Following four rounds of phage selections and initial binding and specificity analyses (Figure S1), four scFvs (scFv-1, 2, 3, and 4) were selected for further characterization (Figure 1). All four scFv fragments were relatively poorly expressed when produced in *E. coli* and, therefore, scFvs were prepared by secretion from Sf9 insect cells, which allowed for higher yields, increased solubility and a reduction in endotoxin contamination (Figures 2A and 2B). The scFvs showed a tendency to dimerize as observed by size-exclusion chromatography (SEC), as well as delayed elution from interaction with the Superdex medium (Figure 2C), which has previously been observed for nanobodies (Zimmermann et al., 2020).

Table 1. Data collection and refinement statistics (molecular replacement)

	6Y6C (scFv-4)	6YMQ (scFv-4)	6YYE (scFv-2)
Data collection			
Space group	P 41 2 2	P 21 21 2	I 2 2 2
Cell dimensions			
a, b, c (Å)	111.66, 111.66, 232.20	167.51, 180.77, 125.51	113.94, 126.20, 225.01
α, β, γ (°)	90, 90, 90	90, 90, 90	90, 90, 90
Resolution (Å)	100.63–2.26 (2.341–2.26)	75.99–3.07 (3.18–3.07)	110.07–3.36 (3.36–3.57)
R_{merge}	0.2129 (2.041)	0.2811 (1.86)	0.266 (1.55)
$I/\sigma I$	9.96 (1.25)	4.2 (1.0)	7.4 (1.7)
Completeness (%)	99.80 (99.34)	99.83 (99.90)	92 (68.9)
Redundancy	21.4 (14.3)	6.4 (6.6)	13.4 (12.7)
Wilson B factor	39.44	69.70	68.7
Refinement			
Resolution (Å)	2.26	3.07	3.36
No. of reflections	69,383 (6,776)	71,714 (7,075)	16,272 (70)
R_{work}/R_{free}	0.191/0.216	0.2730/0.2871	0.298/0.328
No. of atoms			
Protein	5368	15,625	5038
Ligand/ion	28	92	14
Water	375	13	–
B factors			
Protein	42.31	88.15	91.39
Ligand/ion	67.13	90.45	91.5
Water	47.65	58.05	–
RMSD			
Bond lengths (Å)	0.002	0.001	0.003
Bond angles (°)	0.51	0.40	0.56
Solvent (%)	73.85	69	77.18
Average B factor (Å^2)	42.0	88.14	91.0
Anisotropy	0.484	0.641	0.041

RMSD, root-mean-square deviation.

The binding of the scFvs, expressed from Sf9 cells, to TREM2, was characterized by surface plasmon resonance (SPR) to establish their relative binding affinities (Figure S2; apparent $^{App}K_D$ values [Preger et al., 2020] are reported to account for potential avidity effects caused by scFv oligomerization). For these experiments, the human TREM2 antigen was cloned into a mammalian expression vector and produced as a longer biotinylated construct consisting of the Ig domain and stalk region of TREM2 (His19-Ser174) that was then immobilized onto a streptavidin-coated sensor chip. Initial measurements on mixed oligomer samples revealed that all antibody fragments showed strong binding to TREM2 except for scFv-1, which proved to be a weaker binder with an apparent equilibrium dissociation constant ($^{App}K_D$) > 100 nM (Figure S1). The binding kinetics of scFv-3 displayed moderate affinity with a fast dissociation rate ($^{App}K_D$ ~ 54 nM; Figure S2B). By contrast, scFv-2 and scFv-4 were characterized by high-affinity binding, $^{App}K_D$ values of ~1 nM (Figures S2A and S2C), although the accuracy of scFv-4 binding kinetics determination was limited by some non-specific interactions with the SPR sensor chip as well as the possible avidity effects due to scFv oligomerization (Figure 2C).

Structure determination of TREM2-scFv antibody complexes

We hypothesized that scFv antibodies binding at different sites on the TREM2 ectodomain might differentially affect receptor signaling, internalization, or cleavage and, therefore, that an appreciation of their binding sites is important. Hence, we decided to attempt to determine the crystal structures of TREM2 bound to scFv-2, scFv-3, and scFv-4 antibodies whose binding kinetics suggested strong interactions. Crystallization screens were established using the TREM2 Ig domain (His19-Asp131), which has been structurally characterized previously (Kober et al., 2016; Sudom et al., 2018), as well as using the TREM2 Ig domain and stalk (His19-Ser174), which is relevant to shedding by ADAM proteases at H157, but susceptible to degradation *in vitro* due to the inherent flexibility of the C-terminal stalk region (Sudom et al., 2018). Both TREM2 proteins were produced in Expi293F™ human cells in the presence of kifunensine and deglycosylated using Endo H treatment.

Diffracting crystals were first obtained for the complexes of the smaller TREM2 Ig domain with both scFv-2 and scFv-4, enabling their structure determination at 3.36 and 3.07 Å resolution,

CellPress
OPEN ACCESS

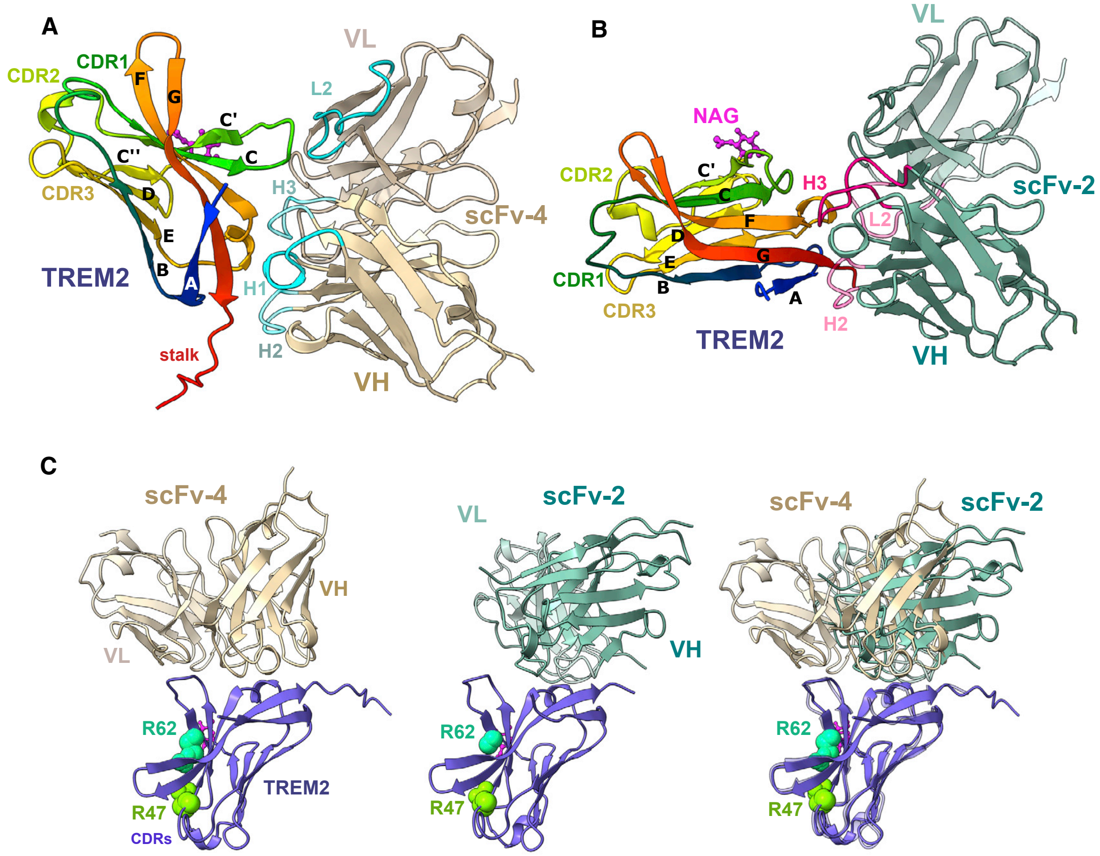

Figure 3. ScFv-2 and scFv-4 bind to partially overlapping epitopes on TREM2 distal to the putative ligand-binding site

(A) Cartoon representation of TREM2 bound to scFv-4. TREM2 β sheets are labeled and colored in rainbow. The TREM2 epitope consists of strand βA (T17-A28), loop C-C′ (R52-P59), and residues in βF (P102-L107) and βG (L125-D131). TREM2 residues in the stalk region could be modeled up to Asp137. ScFv-4 is colored in tan with VH domain positioned on the same plane as VL in lighter tan. ScFv CDR loops involved in TREM2 binding are colored in cyan and labeled. N-Acetyl glucosamine (NAG) remaining after de-glycosylation is colored in pink and shown as ball and stick.

(B) The general topology of the TREM2 and scFv-2 complex. The TREM2 epitope bound by scFv-2 is similar to that of the scFv-4 complex, but has limited interaction with the C-C′ (R52-P59) region. The TREM2 construct crystallized with scFv-2 lacks the stalk sequence and terminates after the βG strand at Asp131. (A) and (B) are fixed on a single antibody orientation resulting in rotation of the TREM2 structures. VH domain of scFv-2 is colored in green and VL domain is colored in light green. ScFv CDR loops involved in TREM2 binding are colored in magenta.

(C) Alternative view showing the structures fixed on a single orientation of TREM2. Superposition of the two complexes highlights differences between the TREM2 epitopes bound by scFv-2 and scFv-4. Genetic variants R47H and R62H are known risk factors for AD (Guerreiro et al., 2013; Jin et al., 2014; Jonsson et al., 2013; Sims et al., 2017). The Arg47 (green) and Arg62 (cyan) side chains are shown in spacefill for reference. Both residues are positioned outside of the complex interfaces.

See also Figures S3–S5.

respectively (see Table 1 for data collection and refinement statistics). Both crystal lattices were characterized by a high solvent content (Figure S3) and, despite screening over 180 crystals, the resolution of the scFv-2 complex could not be improved. Nevertheless, this level of resolution was sufficient to define the scFv-2/TREM2 interface. A higher-resolution structure of the scFv-4 complex was, however, subsequently obtained using the longer TREM2 construct comprising both the Ig domain and stalk region and refined to 2.26 Å resolution. Generally, the scFv-2/TREM2 complex contained fewer crystal contacts than scFv-4, leading to a more spacious lattice and possibly lower resolution (Figure S3). Despite similar efforts, no crystals were obtained for TREM2 in complex with scFv-3. This could reflect its faster dissociation kinetics or perhaps its greater monomer/dimer heterogeneity at high concentrations.

ScFv-2 and scFv-4 bind to partially overlapping epitopes on TREM2 distal to the putative ligand-binding site

Here, we focus our subsequent work on the higher-resolution structure of the scFv-4 complex with the TREM2 Ig domain and stalk (Figure 3A) and the scFv-2 complex with the TREM2 Ig domain alone (Figure 3B). In both structures, the globular Ig domain of TREM2 displayed a β sandwich fold that consisted of nine β strands, including strands A-G common to all Ig-like domains and a C′-C″ insertion typical of the V-set Ig domain. The overall structure does not differ significantly to the previously solved structures of wild-type TREM2 (PDB: 5ELI and 5UD7), except for some variability in the flexible loops (Figure S4A). The putative ligand-binding site on TREM2 is proposed to be located at one end of the β sandwich, which presents the three complementarity-determining regions (CDRs1-3) and includes

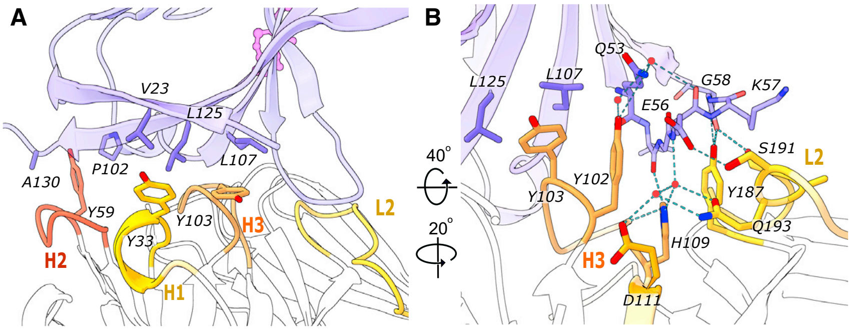

Figure 4. Interactions of scFv-4 with the TREM2 Ig domain

(A) Tyrosine residues of CDR-H1, H2, and H3 intercalating between TREM2 β sheets rich in hydrophobic residues are highlighted as sticks. Interface residues are presented as blue (TREM2) or orange, red, and yellow (scFv-4).

(B) The same structure visualized by rotation as indicated. Residues involved in the hydrogen bond network between CDR-H3, CDR-L2, and TREM2 are presented as sticks. Hydrogen bonds are denoted by a dashed line. Only hydrogen bonds generated by scFv-4 side chains are shown together with water molecules mediating hydrogen bonding when applicable. A large number of contacts are with the TREM2 C-C′ loop. Calculated hydrogen bonds are shorter than 3.5 Å with an angle of 180° ± 10°.

the AD-risk allele sites Arg47, as part of CDR1, and Arg62 in the C′ β strand (Kober et al., 2016; Song et al., 2017; Sudom et al., 2018). Of note, both scFv-2 and scFv-4 were found to bind to the opposite end of TREM2 (Figures 3A–3C). Here, the β sandwich of TREM2 splayed between β strands C and F to provide an extended surface for the scFvs to bind that included parts of β strands A, F, and G, and loop C-C′ (Figures 3A–3C). However, the superposition of the two TREM2-scFv complexes revealed that the binding epitopes on TREM2 were only partially similar (Figure 3C). For instance, scFv-4 formed interactions with the C-C′ loop in TREM2 that were largely absent in the scFv-2 complex. Overall, the structures suggest that the scFvs should find broad application with the binding epitopes being located favorably for scFv interaction with both the ligand-bound and unbound states, as well as mutant states.

Interactions of scFv-4 with the TREM2 ectodomain

Overall, the variable heavy (VH) and variable light (VL) domains of scFv-4 displayed canonical immunoglobulin folds tethered by an engineered glycine-serine-rich linker that was disordered in the refined structure. The binding of scFv-4 to TREM2 was centered on the CDR3 loop of the scFv-4 VH domain (herein CDR-H3), flanked by interactions from CDRs-H1 and H2 and by the VL domain CDR-L2 (Figures 4A and 4B).

The resolution of the scFv-4 complex structure enabled a detailed analysis of the side chain interactions in the binding interface (Figures 4A and 4B). A striking feature of the CDR sequence selections in scFv-4 was the enrichment of tyrosine residues (Figure 1). Indeed, CDR-H3 contained a sequence of six consecutive tyrosines, interrupted only by Gly104 (numbering corresponding to PDB entry sequences), which was likely selected to avoid the steric clashes that would result from any other amino acid at this position. Tyrosine residues 33, 59, 102, 103, 106, 107, and 187 in scFv-4 contributed to extensive hydrophobic packing interactions with the non-polar TREM2 residues Val23, Pro102, Leu107, Leu125, Val128, and Ala130 (Figures 4A and 4B). Additional side chain- and water-mediated hydrogen bond interactions were provided by scFv-4 loops CDR-H3 and CDR-L2 (Figure 4B). The most extensive hydrogen bond interactions occurred between the CDR-L2 with the C-C′ loop of TREM2. Here, the scFv-4 residues Tyr187, Ser191, and Ser194 formed hydrogen bonds with TREM2 residues Lys47, Gly58, and Glu56, respectively (Figure 4B). Tyr102 from CDR-H3 also inserted between TREM2 β strands C and F to form a further hydrogen bond with TREM2 Gln53 (Figure 4B). No significant differences were observed in scFv-4 binding to the TREM2 Ig domain in the equivalent structure using the shorter TREM2 construct His19-Asp131 (Figure S4B).

While the primary scFv-4 interaction was mediated by the TREM2 Ig domain as described above, we identified crystal contacts between the TREM2 stalk and CDR-H1 from a neighboring scFv-4 subunit. This additional packing allowed the stalk to be traced in the electron density maps up to Asp137 in the C terminus and several hydrogen bond interactions were observed to support the stalk interaction (Figure S5).

Interactions of scFv-2 with the TREM2 ectodomain

The binding of scFv-2 to TREM2 was mediated by the three CDR loops of the VH domain (CDRs-H1, H2, and H3), as well as by CDR-L3 from the light domain (Figures 5A and 5B). Overall, these loops were enriched in tyrosine and serine residues that contributed to a mix of hydrophobic and hydrogen bond interactions. Of note, TREM2 Glu127 (βG strand) was within hydrogen bonding distance of three scFv-2 side chains, including Ser54, Ser56, and Ser59, from CDR-H2. CDR-H2 Ser59 was additionally within hydrogen bonding distance of TREM2 Gln25 (βA). The sole glycine in CDR-L3 (Gly231) also contributed a hydrogen bond from its carbonyl group to the side chain of TREM2 His103.

Together the scFv-2 and scFv-4 complex structures show the potential value of these antibody fragments as crystallization chaperones. More importantly, however, they define the epitopes of these scFvs, allowing for the interpretation of functional properties in the context of the binding site on the surface of TREM2, which has been poorly described for other anti-TREM2 antibodies.

ScFv-3 and scFv-4 reduce shedding of sTREM2 from HEK293 cells overexpressing TREM2 and DAP12

The H157Y polymorphism, which leads to accelerated cleavage of TREM2 and increases AD risk, suggests that regulation of shedding of sTREM2 might be important in human biology (Jiang et al., 2016; Thornton et al., 2017). Based on the potential interactions of scFv-4 with the stalk domain, as indicated by the crystal contacts, and the potential for TREM2 internalization induced by scFv dimers, we decided to determine the effect of the scFv antibodies on sTREM2 shedding. We devised a sandwich ELISA, capturing

CellPress
OPEN ACCESS

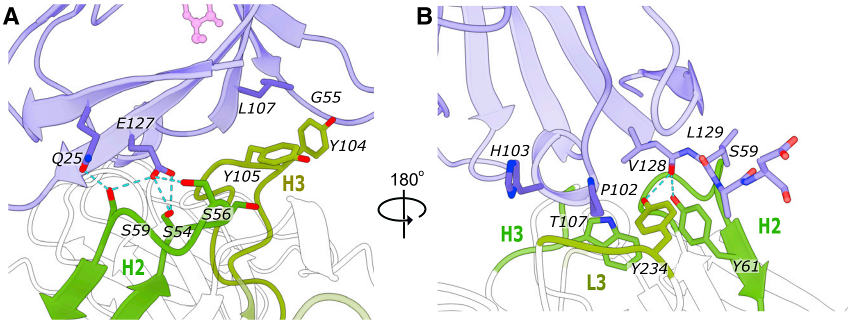

Figure 5. Interactions of scFv-2 with the TREM2 Ig domain

(A) Residues involved in hydrogen bonding and hydrophobic packing are represented as sticks and shown in blue (TREM2) or green (scFv-2). The aliphatic side chains of tyrosines 104 and 105 in CDR-H3 pack against the backbone of the C-C′ loop (Gly55) and Leu107 of TREM2. The serine-rich CDR-H2 mediates multiple hydrogen bonds with the side chains of TREM2 Glu127 and Gln25.

(B) 180°C rotated view of the interface between TREM2 and scFv-2. Hydrogen bonds are observed between the hydroxyl groups of Tyr234 (L3) and Tyr61 (H2) and the carbonyl of TREM2 Val128 and are depicted by a blue dashed line. Further hydrophobic packing is mediated by Trp107 (H3) and other side chains from CDR-H2 and CDR-L3.

sTREM2 from cell culture supernatants, and confirmed that scFvs were not masking the sTREM2 epitopes required by the ELISA capture and detection antibodies (Figure S6). Recently another metalloprotease, meprin β, was shown to cleave TREM2, with the main cleavage site between Arg136 and Asp137 (Berner et al., 2020). We confirmed that both the Ig domain as well as a region of the stalk C-terminal to Ala138 were required for detection in our ELISA protocol. Thus, the detected sTREM2 product is unlikely to be resulting from meprin β cleavage (Figure S6). Batimastat, a broad-spectrum chemical inhibitor of matrix metalloproteinases, including ADAM proteases, was used as a positive control to inhibit shedding. HEK293 cells stably transfected with TREM2 and DAP12 were treated with each of the scFvs for 5 h to reach the appropriate threshold for sTREM2 detection in the supernatant. ScFv-3 and scFv-4 (10 μg/mL) significantly reduced shedding of sTREM2 by 22% and 38%, respectively (Figure 6A).

Oligomerization of scFvs has been observed and can be exploited to enhance the properties of selected molecules (Kortt et al., 2001). We decided to investigate the effect on shedding of the apparent monomer and dimer species present in our scFv-3 and scFv-4 samples. After these species were acutely separated by SEC and processed rapidly to minimize re-equilibration between low- and high-molecular-weight (MW) species, it was found that the monomer species for both scFv-3 or scFv-4 had minimal or no activity (Figure 6B), whereas the dimers decreased production of sTREM2 by more than 50% (Figure 6B). Concentration-response experiments for the scFv-3 and scFv-4 dimers showed that maximum effect plateaued between 50% and 70% with calculated half-maximal effective concentration values of 2.5 and 3 nM, respectively (Figures 6C and 6D). Of note, scFv-2, which showed a high binding affinity for TREM2, caused no changes in sTREM2 release despite having a similar binding epitope to scFv-4 (Figure 6A). This antibody fragment purified mostly as a monomer, further highlighting the importance of scFv oligomerization for reduction of shedding. None of the scFvs impacted cell viability, as measured by counting cell nuclei using Hoechst staining and fluorescence-based viability (data not shown).

Finally, to demonstrate selective binding to TREM2, we conjugated the dimeric fractions of scFv-3 and scFv-4 to Alexa Fluor 647 and repeated the HEK293 treatment protocol with subsequent imaging of the fluorescent scFvs (Figure 7). Labeling of the cells was dependent upon the heterologous expression of TREM2/DAP12. Most of the Alexa signal was present intracellularly and was resistant to acidic wash, consistent with TREM2-mediated internalization under the conditions tested. ScFv-4 appeared to show superior specificity compared with scFv-3 based on the staining of non-transfected HEK293 cells (Figure 7).

DISCUSSION

In this paper, we have presented our work on the structural characterization of scFvs raised against human TREM2 by phage display. We screened a library of scFv antibody fragments against fully folded and glycosylated TREM2 Ig domain, which resulted in the generation of three successful candidates. Despite challenging purification procedures, we were successful in producing recombinant antibody fragments and demonstrated them to be suitable for X-ray crystallography. We have solved the structures of two scFvs bound to TREM2 and provide the first crystallographic structures of antibody interactions with TREM2. These are also the first antibody fragments reported to unambiguously bind to the TREM2 globular Ig domain. In addition, we have shown that higher-MW oligomeric species of scFvs are able to display enhanced functional activity, suggesting parallels with the activity of bivalent mAbs and diabodies.

Since TREM2 is a potential therapeutic target for AD, the pharmaceutical industry has initiated several projects aimed at developing TREM2 antibody therapeutics, with at least two candidates having commenced clinical trials. Data have been reported for two, AL002 and 4D9, which allow for an interesting comparison with scFv-4 (Schlepckow et al., 2020; Wang et al., 2020). Other functionally active antibodies have either not been widely published (WO2016023019A2) or the epitope has not been defined and, therefore, are not included in the discussion below (Cheng et al., 2018). X-ray crystallographic data, which accurately define the antibody/TREM2 interfaces, are not publicly available for any of these reagents. AL002 is an agonist antibody with a poorly defined epitope most likely in the stalk region between residues 112 and 174, while 4D9 has an epitope defined by peptide mapping to residues Asp137-Gly145 (Schlepckow et al., 2020; Wang et al., 2020). As such, they target TREM2 epitopes N-terminal of the α-secretase cleavage site at H157, whereas our X-ray crystallography data show that scFv-4 binds the Ig domain of TREM2. The binding sites do show some cross-species variation, explaining the observation that 4D9 is specific

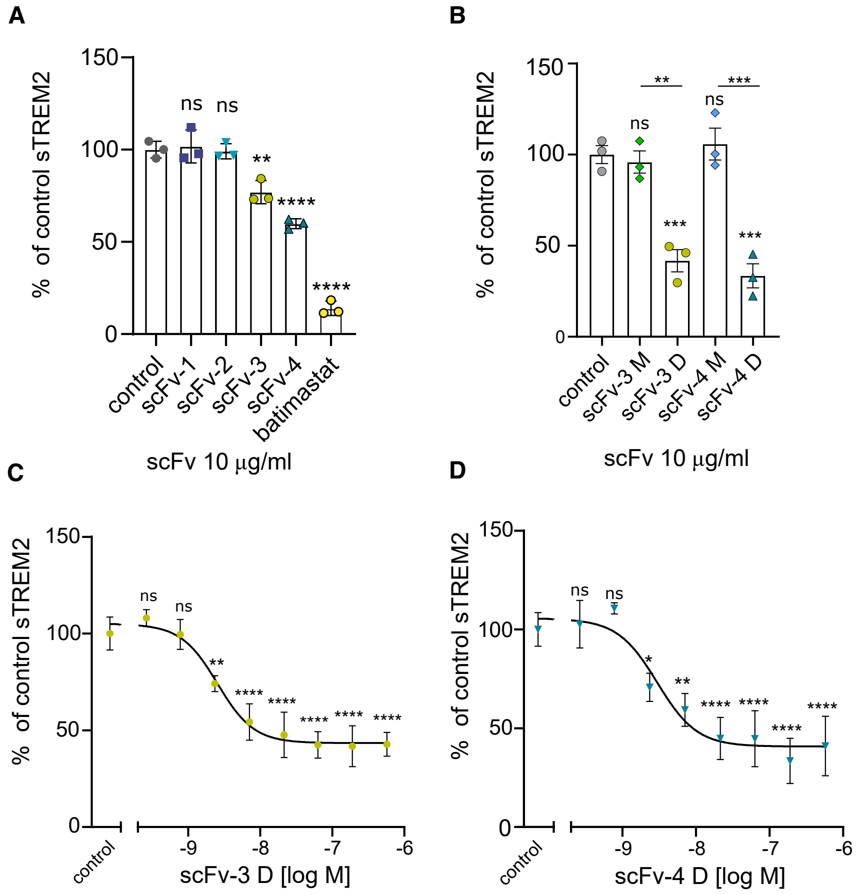

Figure 6. ScFv-3 and scFv-4 and their oligomers reduce TREM2 shedding in HEK293 cells overexpressing TREM2 and DAP12

(A) HEK293 cells overexpressing TREM2 and DAP12 were treated with scFvs at 10 µg/mL for 5 h or with 20 µM batimastat or buffer in control samples. Data were normalized to cell number by nuclei counting and represent the mean ± SD (n = 3) as percent reduction of sTREM2 detected in buffer only sample (control). One-way ANOVA, Dunnett's post hoc test comparison with buffer only control was carried out with significant p values scFv-3 versus control = 0.0011, scFv-4 versus control <0.0001, batimastat versus control <0.0001.

(B) Results of shedding assay described above for separated scFv monomers (M) or dimers (D). ELISA results were normalized to cell density. One-way ANOVA, Tukey's post hoc test comparison with significant p values scFv-3 (D) versus control = 0.0007, scFv-4 (D) versus control = 0.0002, scFv-3 monomer versus dimer <0.0013, scFv-4 monomer versus dimer <0.0004, sTREM2 in batimastat samples were below detection threshold.

(C and D) (C) Assay of scFv-3 dimer showing scFv-concentration-dependent reduction in shedding. Data represent the mean ± SD (n = 3) of independent experiments, one-way ANOVA, Dunnett's post hoc comparison with buffer control, test $p < 0.0001$, half-maximal effective concentration (EC_{50}) = 2.5 nM. (D) scFv-4 dimer concentration-dependent reduction in shedding. ELISA results were normalized to cell density. Data represent the mean ± SD (n = 3) of independent experiments, One-way ANOVA, Dunnett's post hoc comparison with buffer control, test $p < 0.0001$, EC_{50} = 3 nM.

See also Figure S6.

for mouse TREM2, whereas AL002 has a high affinity for human TREM2.

As both AL002 and 4D9 are stalk binding antibodies, it is possible that they might affect cleavage of TREM2 by virtue of sterically hindering access by ADAMs. Consistent with that hypothesis, both full-length antibodies (4D9 and AL002) have been shown to reduce shedding of sTREM2. This interesting property has been confirmed for the first time in human with AL002 (Wang et al., 2020). This function also appears to be dependent upon the use of a bivalent antibody, as the 4D9 Fab fails to inhibit shedding of sTREM2 by cells. These observations are in agreement with our data, where higher-MW oligomers of scFv-3 and scFv-4 inhibit up to 70% of sTREM2 production.

At present, the mechanism of action for the inhibition of shedding by all of these antibody reagents is unclear. It might occur either by inhibition of cleavage of TREM2 at the cell surface or by promoting internalization of full-length TREM2, thereby removing substrate availability. Schlepckow et al. propose that 4D9 exerts some of its effects by stabilizing TREM2 at the plasma membrane, explaining the potentiation of signaling in response to liposomes by 4D9. However, it is also possible that reduction of sTREM2 production by bivalent antibodies or oligomeric scFvs is due to crosslinking of receptor and internalization, thereby reducing cell surface substrate for shedding. It is noteworthy that a bivalent antibody is necessary for 4D9 functional effects and, to date, we have not observed any functional responses to monomeric scFv-4, only observing activity in preparations containing appreciable dimeric species. Furthermore, Alexa Fluor-labeled scFv-4 and scFv-3 label HEK293 cells in a TREM2 expression-dependent manner, leading to intracellular immunofluorescence. Interestingly, the scFv-2 antibody fragment has the lowest propensity to form oligomeric species and did not show any inhibitory activity in the sTREM2 shedding assay. Our data are therefore consistent with the effect of scFv-3 and scFv-4 upon shedding of sTREM2 being due to promoting the internalization of TREM2.

Both AL002 and 4D9 show agonist properties *in vitro*. AL002 elicits TREM2-mediated secondary intracellular signaling, demonstrated using an NFAT reporter cell line and phosphorylation of a protein of equal MW to DAP12 in bone-derived macrophages (Wang et al., 2020). Similarly, 4D9 shows agonism, but only when a full-length antibody was used to challenge cells; Syk phosphorylation did not occur following exposure of cells to a 4D9 Fab fragment. The requirement for a bivalent antibody for the function of AL002 has not been reported, but these observations with 4D9 led the authors to hypothesize that the functional effects of 4D9 are dependent upon a bivalent antibody and crosslinking of TREM2 molecules (Schlepckow et al., 2020). Taken together, the literature and our own data highlight the importance of multivalency for the functional activities of anti-TREM2 antibodies. On account of the need to prepare bivalent mAb and Fab antibodies, a full investigation of the agonist

CellPress
OPEN ACCESS

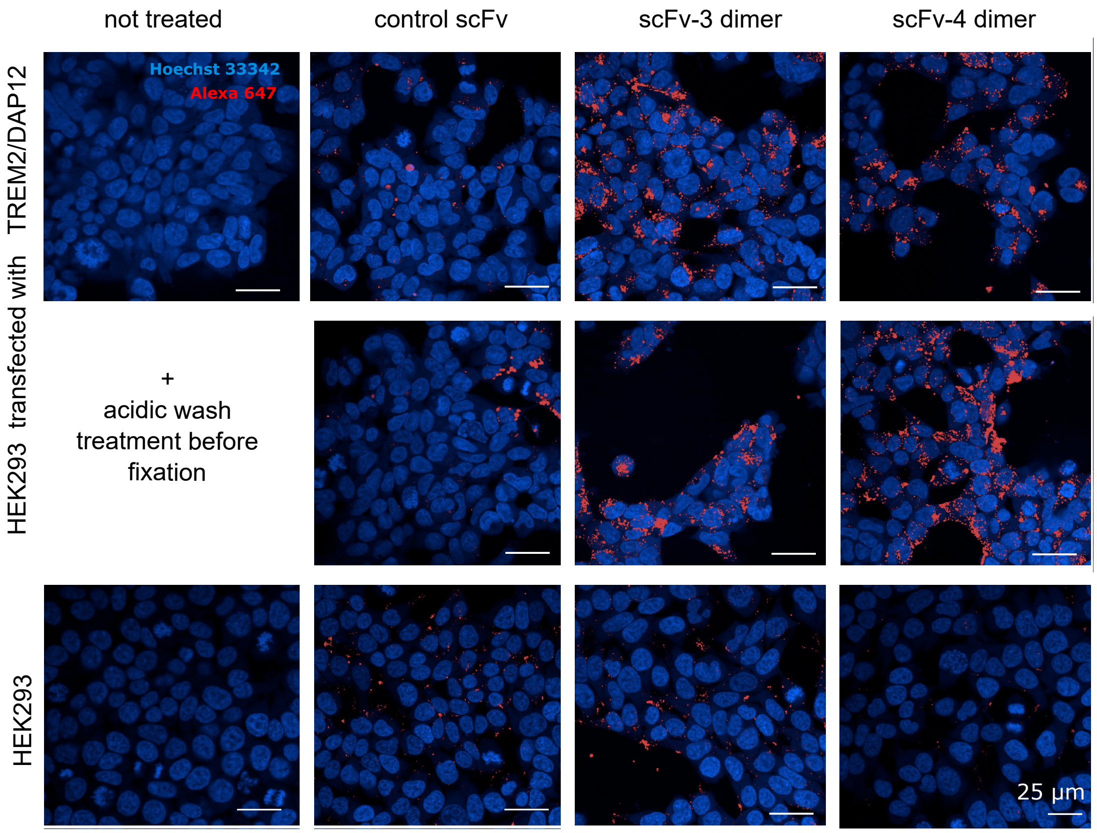

Figure 7. ScFv-3 and 4 engage TREM2 in HEK293 cells overexpressing TREM2/DAP12 and produce intracellular signal after 5 h treatment
HEK293 cells transfected with TREM2/DAP12 or wild-type HEK293 were incubated with dimers of scFv-3 and scFv-4 or control scFvs against different target pre-conjugated to Alexa Fluor 647 or buffer for 5 h. Cells were washed with medium or DMEM, pH 2, medium twice before fixation and staining. Nuclei were visualized by Hoechst staining (blue) and scFvs by Alexa Fluor 647 (red) using an Opera Phenix confocal lens with 40× magnification, n = 1 with 3 technical repeats. Scale bars, 25 μm.

and antagonist properties of the scFvs obtained in our studies is the subject of a separate piece of work.

While we have presented evidence that scFvs capable of dimerization reduce sTREM2 shedding from the HEK293 cells, the functional impact of monomeric scFvs is unclear due to the dynamic equilibrium between monomeric and dimeric forms. However, we have observed a decrease in activity upon storage of a purified fraction of dimers and also negligible activity of freshly prepared and rapidly utilized monomers, suggesting that the monomer represents an inactive conformation (data not shown). Similarly to the mAbs discussed above, multivalent scFvs will have the potential to crosslink TREM2 ectodomains into higher-order structures, thereby creating significant steric hindrance to proteolytic complexes, altering their characteristics as substrates, or triggering endocytosis.

By using phage display to raise recombinant scFvs we have assured that the source of the molecules is renewable and can be easily shared. During our studies, we have encountered difficulties caused by the use of commercially available antibodies with unknown TREM2 epitopes, especially when used in ELISA, pull-down studies or immunocytochemistry. The presented scFvs bind away from the proposed ligand recognition site, qualifying them for development into additional tools for the study of TREM2-ligand interactions and receptor processing.

Our data represent the first crystallographic characterization of binding interactions between functional antibody fragments and TREM2 and we hope that these additional tools will help elucidate the complex pathophysiology of TREM2 in AD.

STAR★METHODS

Detailed methods are provided in the online version of this paper and include the following:

- KEY RESOURCES TABLE
- RESOURCE AVAILABILITY
  - Lead contact
  - Materials availability
  - Data and code availability
- EXPERIMENTAL MODEL AND SUBJECT DETAILS
  - Cell lines
- METHOD DETAILS
  - Cloning of TREM2 and scFvs
  - Expression and purification of TREM2 and scFvs in baculovirus expression system
  - Expression and purification of TREM2 in mammalian cells
  - Antibody selection and validation

  - ScFv binding kinetics
  - Crystallisation
  - Data collection and structure determination
  - Mammalian cell culture for cell biology
  - sTREM2 ELISA
  - Alexa fluor 647 cell staining and imaging
- QUANTIFICATION AND STATISTICAL ANALYSIS

SUPPLEMENTAL INFORMATION

Supplemental information can be found online at https://doi.org/10.1016/j.str.2021.06.010.

ACKNOWLEDGMENTS

The authors thank Diamond Light Source for beamtime (proposal mx15433), as well as the staff of beamline I24 for assistance with crystal testing and data collection. We would like to acknowledge Prof. Peter St George-Hyslop for the gift of TREM2-DAP12-expressing HEK293 cells and valuable advice, Andy Takle, James Staddon, Karel Otero-Gutierrez, and Suresh Poda for advice during the project. The SGC is a registered charity (no. 1097737) that receives funds from AbbVie, Bayer Pharma AG, Boehringer Ingelheim, Canada Foundation for Innovation, Eshelman Institute for Innovation, Genome Canada, Innovative Medicines Initiative (EU/EFPIA) (ULTRA-DD grant no. 115766), Janssen, Merck KGaA Darmstadt Germany, MSD, Novartis Pharma AG, Ontario Ministry of Economic Development and Innovation, Pfizer, São Paulo Research Foundation-FAPESP, Takeda, and Wellcome Trust (106169/ZZ14/Z). A.S. was supported by a DPhil studentship sponsored by Eisai. J.B.D., E.M., T.B.S., and E.DiD. were supported by a grant from Alzheimer's Research UK (ARUK-2015DDI-OX).

AUTHOR CONTRIBUTIONS

Conceptualization, A.S., J.B.D., N.A.B.-B., and A.N.B.; provision of resources, Y.C., J.Y., D.Q., S.M.M., S.G., P.J.A., and J.W.; investigation, A.S., J.Y., D.Q., C.P., and E.W.; supervision, Y.C., T.B.S., S.G., H.P., P.J.A., E.DiD., E.M., J.B.D., N.A.B.-B., and A.N.B.; manuscript preparation, A.S., C.P., S.G., E.DiD., J.B.D., N.A.B.-B., and A.N.B.; funding acquisition, J.W. and J.B.D. All authors reviewed the manuscript.

DECLARATION OF INTERESTS

Y.C., S.J.N., P.J.A., and J.W. are employees of Eisai Ltd., and Eisai Inc.

Received: January 29, 2021
Revised: May 11, 2021
Accepted: June 9, 2021
Published: July 6, 2021

REFERENCES

Aricescu, A.R., Lu, W., and Jones, E.Y. (2006). A time- and cost-efficient system for high-level protein production in mammalian cells. Acta Crystallogr. D. Biol. Crystallogr. *62*, 1243–1250.

Berner, D.K., Wessolowski, L., Armbrust, F., Schneppenheim, J., Schlepckow, K., Koudelka, T., Scharfenberg, F., Lucius, R., Tholey, A., Kleinberger, G., et al. (2020). Meprin β cleaves TREM2 and controls its phagocytic activity on macrophages. FASEB J. *34*, 6675–6687.

Block, M.L., Zecca, L., and Hong, J.S. (2007). Microglia-mediated neurotoxicity: uncovering the molecular mechanisms. Nat. Rev. Neurosci. *8*, 57–69.

Bricogne, G., Blanc, E., Brandl, M., Flensburg, C., Keller, P., Paciorek, W., Roversi, P., Sharff, A., Smart, O.S., Vonrhein, C., et al. (2016). BUSTER Version X.Y.Z. Cambridge, United Kingdom Glob (Phasing Ltd).

Cheng, Q., Danao, J., Talreja, S., Wen, P., Yin, J., Sun, N., Li, C.M., Chui, D., Tran, D., Koirala, S., et al. (2018). TREM2-activating antibodies abrogate the negative pleiotropic effects of the Alzheimer's disease variant Trem2R47Hon murine myeloid cell function. J. Biol. Chem. *293*, 12620–12633.

Ellwanger, D.C., Wang, S., Brioschi, S., Shao, Z., Green, L., Case, R., Yoo, D., Weishuhn, D., Rathanaswami, P., Bradley, J., et al. (2021). Prior activation state shapes the microglia response to antihuman TREM2 in a mouse model of Alzheimer's disease. Proc. Natl. Acad. Sci. *118*, e2017742118.

Emsley, P., Lohkamp, B., Scott, W.G., and Cowtan, K. (2010). Features and development of coot. Acta Crystallogr. D Biol. Crystallogr. *75*, 861–877.

Evans, P. (2006). Scaling and assessment of data quality. Acta Crystallogr. D Biol. Crystallogr. *62*, 72–82.

Fairhead, M., Preger, C., Wigren, E., Strain-Damerell, C., Ossipova, E., Ye, M., Makola, M., Burgess-Brown, N.A., Persson, H., von Delft, F., et al. (2019). Strategies to improve scFvs as crystallization chaperones suggested by analysis of a complex with the human PHD-bromodomain SP140. bioRxiv, 767376. https://doi.org/10.1101/767376.

Fassler, M., Rappaport, M.S., Cuño, C.B., and George, J. (2021). Engagement of TREM2 by a novel monoclonal antibody induces activation of microglia and improves cognitive function in Alzheimer's disease models. J. Neuroinflammation *18*, 1–18.

Goddard, T.D., Huang, C.C., Meng, E.C., Pettersen, E.F., Couch, G.S., Morris, J.H., and Ferrin, T.E. (2018). UCSF ChimeraX: meeting modern challenges in visualization and analysis. Protein Sci. *27*, 14–25.

Guerreiro, R., Wojtas, A., Bras, J., Carrasquillo, M., Rogaeva, E., Majounie, E., Cruchaga, C., Sassi, C., Kauwe, J.S.K., Younkin, S., et al. (2013). *TREM2* variants in Alzheimer's disease. N. Engl. J. Med. *368*, 117–127.

Ibach, M., Mathews, M., Linnartz-Gerlach, B., Theil, S., Kumar, S., Feederle, R., Brüstle, O., Neumann, H., and Walter, J. (2021). A reporter cell system for the triggering receptor expressed on myeloid cells 2 reveals differential effects of disease-associated variants on receptor signaling and activation by antibodies against the stalk region. Glia *69*, 1126–1139.

Jay, T.R., Hirsch, A.M., Broihier, M.L., Miller, C.M., Neilson, L.E., Ransohoff, R.M., Lamb, B.T., and Landreth, G.E. (2017). Disease progression-dependent effects of TREM2 deficiency in a mouse model of Alzheimer's disease. J. Neurosci. *37*, 637–647.

Jiang, T., Tan, L., Chen, Q., Tan, M.S., Zhou, J.S., Zhu, X.C., Lu, H., Wang, H.F., Zhang, Y.D., and Yu, J.T. (2016). A rare coding variant in TREM2 increases risk for Alzheimer's disease in Han Chinese. Neurobiol. Aging *42*, 217.e1–217.e3.

Jin, S.C., Benitez, B.A., Karch, C.M., Cooper, B., Skorupa, T., Carrell, D., Norton, J.B., Hsu, S., Harari, O., Cai, Y., et al. (2014). Coding variants in TREM2 increase risk for Alzheimer's disease. Hum. Mol. Genet. *23*, 5838–5846.

Jonsson, T., Stefansson, H., Steinberg, S., Jonsdottir, I., Jonsson, P.V., Snaedal, J., Bjornsson, S., Huttenlocher, J., Levey, A.I., Lah, J.J., et al. (2013). Variant of TREM2 associated with the risk of Alzheimer's disease. N. Engl. J. Med. *368*, 107–116.

Kabsch, W., T., B.A., K., D., A., K.P., K., D., S., M., G., R.R.B., P., E., S., F., K., W., et al. (2010). XDS. Acta Crystallogr. D Biol. Crystallogr. *66*, 125–132.

Keren-Shaul, H., Spinrad, A., Weiner, A., Matcovitch-Natan, O., Dvir-Szternfeld, R., Ulland, T.K., David, E., Baruch, K., Lara-Astaiso, D., Toth, B., et al. (2017). A unique microglia type associated with restricting development of Alzheimer's disease. Cell *169*, 1276–1290.e17.

Kober, D.L., Alexander-Brett, J.M., Karch, C.M., Cruchaga, C., Colonna, M., Holtzman, M.J., and Brett, T.J. (2016). Neurodegenerative disease mutations in TREM2 reveal a functional surface and distinct loss-of-function mechanisms. eLife *5*, e20391.

Konishi, H., and Kiyama, H. (2018). Microglial TREM2/DAP12 signaling: a double-edged sword in neural diseases. Front. Cell. Neurosci. *12*, 1–14.

Kortt, A.A., Dolezal, O., Power, B.E., and Hudson, P.J. (2001). Dimeric and trimeric antibodies: high avidity scFvs for cancer targeting. Biomol. Eng. *18*, 95–108.

Krasemann, S., Madore, C., Cialic, R., Baufeld, C., Calcagno, N., El Fatimy, R., Beckers, L., O'Loughlin, E., Xu, Y., Fanek, Z., et al. (2017). The TREM2-APOE

CellPress
OPEN ACCESS

pathway drives the transcriptional phenotype of dysfunctional microglia in neurodegenerative diseases. Immunity *47*, 566–581.e9.

Lambert, J.C., Ibrahim-Verbaas, C.A., Harold, D., Naj, A.C., Sims, R., Bellenguez, C., Jun, G., DeStefano, A.L., Bis, J.C., Beecham, G.W., et al. (2013). Meta-analysis of 74,046 individuals identifies 11 new susceptibility loci for Alzheimer's disease. Nat. Genet. *45*, 1452–1458.

Lefranc, M.-P., Pommié, C., Ruiz, M., Giudicelli, V., Foulquier, E., Truong, L., Thouvenin-Contet, V., and Lefranc, G. (2003). IMGT unique numbering for immunoglobulin and T cell receptor variable domains and Ig superfamily V-like domains. Dev. Comp. Immunol. *27*, 55–77.

Liebschner, D., Afonine, P.V., Baker, M.L., Bunkoczi, G., Chen, V.B., Croll, T.I., Hintze, B., Hung, L.W., Jain, S., McCoy, A.J., et al. (2019). Macromolecular structure determination using X-rays, neutrons and electrons: recent developments in Phenix. Acta Crystallogr. D Struct. Biol. *75*, 861–877.

Long, J.M., and Holtzman, D.M. (2019). Alzheimer disease: an update on pathobiology and treatment strategies. Cell *179*, 312–339.

Nugent, A.A., Lin, K., van Lengerich, B., Lianoglou, S., Przybyla, L., Davis, S.S., Llapashtica, C., Wang, J., Kim, D.J., Xia, D., et al. (2020). TREM2 regulates microglial cholesterol metabolism upon chronic phagocytic challenge. Neuron *105*, 837–854.e9.

Paloneva, J., Mandelin, J., Kiialainen, A., Böhling, T., Prudlo, J., Hakola, P., Haltia, M., Konttinen, Y.T., and Peltonen, L. (2003). DAP12/TREM2 deficiency results in impaired osteoclast differentiation and osteoporotic features. J. Exp. Med. *198*, 669–675.

Parhizkar, S., Arzberger, T., Brendel, M., Kleinberger, G., Deussing, M., Focke, C., Nuscher, B., Xiong, M., Ghasemigharagoz, A., Katzmarski, N., et al. (2019). Loss of TREM2 function increases amyloid seeding but reduces plaque-associated ApoE. Nat. Neurosci. *22*, 191–204.

Piccio, L., Buonsanti, C., Cella, M., Tassi, I., Schmidt, R.E., Fenoglio, C., Rinker, J.2nd, Naismith, R.T., Panina-Bordignon, P., Passini, N., et al. (2008). Identification of soluble TREM-2 in the cerebrospinal fluid and its association with multiple sclerosis and CNS inflammation. Brain *131*, 3081–3091.

Preger, C., Wigren, E., Ossipova, E., Marks, C., Lengqvist, J., Hofström, C., Andersson, O., Jakobsson, P.-J., Gräslund, S., and Persson, H. (2020). Generation and validation of recombinant antibodies to study human aminoacyl-tRNA synthetases. J. Biol. Chem. *295*, 13981–13993.

Price, B.R., Sudduth, T.L., Weekman, E.M., Johnson, S., Hawthorne, D., Woolums, A., and Wilcock, D.M. (2020). Therapeutic Trem2 activation ameliorates amyloid-beta deposition and improves cognition in the 5XFAD model of amyloid deposition. J. Neuroinflammation *17*, 1–13.

Rauchmann, B.S., Schneider-Axmann, T., Alexopoulos, P., and Perneczky, R. (2019). CSF soluble TREM2 as a measure of immune response along the Alzheimer's disease continuum. Neurobiol. Aging *74*, 182–190.

Säll, A., Walle, M., Wingren, C., Müller, S., Nyman, T., Vala, A., Ohlin, M., Borrebaeck, C.A.K., and Persson, H. (2016). Generation and analyses of human synthetic antibody libraries and their application for protein microarrays. Protein Eng. Des. Sel. *29*, 427–437.

Schlepckow, K., Kleinberger, G., Fukumori, A., Feederle, R., Lichtenthaler, S.F., Steiner, H., and Haass, C. (2017). An Alzheimer-associated TREM2 variant occurs at the ADAM cleavage site and affects shedding and phagocytic function. EMBO Mol. Med. *9*, 1356–1365.

Schlepckow, K., Monroe, K.M., Kleinberger, G., Cantuti-Castelvetri, L., Parhizkar, S., Xia, D., Willem, M., Werner, G., Pettkus, N., Brunner, B., et al. (2020). Enhancing protective microglial activities with a dual function TREM2 antibody to the stalk region. EMBO Mol. Med. *12*, e11227.

Shi, Y., and Holtzman, D.M. (2018). Interplay between innate immunity and Alzheimer disease: APOE and TREM2 in the spotlight. Nat. Rev. Immunol. *18*, 759–772.

Sims, R., van der Lee, S.J., Naj, A.C., Bellenguez, C.C., Badarinarayan, N., Jakobsdottir, J., Kunkle, B.W., Boland, A., Raybould, R., Bis, J.C., et al. (2017). Rare coding variants in PLCG2, ABI3, and TREM2 implicate microglial-mediated innate immunity in Alzheimer's disease. Nat. Genet. *49*, 1373–1384.

Song, W., Hooli, B., Mullin, K., Jin, S.C., Cella, M., Ulland, T.K., Wang, Y., Tanzi, R.E., and Colonna, M. (2017). Alzheimer's disease-associated TREM2 variants exhibit either decreased or increased ligand-dependent activation. Alzheimer's Dement. *13*, 381–387.

Strain-Damerell, C., Mahajan, P., Gileadi, O., and Burgess-Brown, N.A. (2014). Medium-throughput production of recombinant human proteins: ligation-independent cloning. In Structural Genomics: General Applications, Y.W. Chen, ed. (Humana Press)), pp. 55–72.

Suárez-Calvet, M., Caballero, M.Á.A., Kleinberger, G., Bateman, R.J., Fagan, A.M., Morris, J.C., Levin, J., Danek, A., Ewers, M., and Haass, C. (2016). Early changes in CSF sTREM2 in dominantly inherited Alzheimer's disease occur after amyloid deposition and neuronal injury. Sci. Transl. Med. *8*, 34–38.

Sudom, A., Talreja, S., Danao, J., Bragg, E., Kegel, R., Min, X., Marcora, E., Thibault, S., Bradley, J., Wood, S., et al. (2018). Molecular basis for the loss-of-function effects of the Alzheimer's disease-associated R47H variant of the immune receptor TREM2 *293*, 12634–12646.

Thornton, P., Sevalle, J., Deery, M.J., Fraser, G., Zhou, Y., Ståhl, S., Franssen, E.H., Dodd, R.B., Qamar, S., Gomez Perez-Nievas, B., et al. (2017). TREM2 shedding by cleavage at the H157-S158 bond is accelerated for the Alzheimer's disease-associated H157Y variant. EMBO Mol. Med. *9*, e201707673.

Ulland, T.K., and Colonna, M. (2018). TREM2—a key player in microglial biology and Alzheimer disease. Nat. Rev. Neurol. *14*, 667–675.

Ulland, T.K., Song, W.M., Huang, S.C.-C.C., Ulrich, J.D., Sergushichev, A., Beatty, W.L., Loboda, A.A., Zhou, Y., Cairns, N.J., Kambal, A., et al. (2017). TREM2 maintains microglial metabolic fitness in Alzheimer's disease. Cell *170*, 649–663.e13.

Ulrich, J.D., and Holtzman, D.M. (2017). Elucidating the role of TREM2 in Alzheimer's disease. Neuron *94*, 237–248.

Vonrhein, C., Flensburg, C., Keller, P., Sharff, A., Smart, O., Paciorek, W., Womack, T., and Bricogne, G. (2011). Data processing and analysis with the autoPROC toolbox. Acta Crystallogr. D Biol. Crystallogr. *67*, 293–302.

Vonrhein, C., Tickle, I.J., Flensburg, C., Keller, P., Paciorek, W., Sharff, A., and Bricogne, G. (2018). Advances in automated data analysis and processing within autoPROC, combined with improved characterisation, mitigation and visualisation of the anisotropy of diffraction limits using STARANISO. Acta Crystallogr. A *74*, a360.

Wang, S., Mustafa, M., Yuede, C.M., Salazar, S.V., Kong, P., Long, H., Ward, M., Siddiqui, O., Paul, R., Gilfillan, S., et al. (2020). Anti-human TREM2 induces microglia proliferation and reduces pathology in an Alzheimer's disease model. J. Exp. Med. *217*, e20200785.

Wang, Y., Cella, M., Mallinson, K., Ulrich, J.D., Young, K.L., Robinette, M.L., Gilfillan, S., Krishnan, G.M., Sudhakar, S., Zinselmeyer, B.H., et al. (2015). TREM2 lipid sensing sustains the microglial response in an Alzheimer's disease model. Cell *160*, 1061–1071.

Winter, G. (2010). Xia2: an expert system for macromolecular crystallography data reduction. J. Appl. Crystallogr. *43*, 186–190.

Winter, G., Waterman, D.G., Parkhurst, J.M., Brewster, A.S., Gildea, R.J., Gerstel, M., Fuentes-Montero, L., Vollmar, M., Michels-Clark, T., Young, I.D., et al. (2018). DIALS: implementation and evaluation of a new integration package. Acta Crystallogr. D Struct. Biol. *74*, 85–97.

Wunderlich, P., Glebov, K., Kemmerling, N., Tien, N.T., Neumann, H., and Walter, J. (2013). Sequential proteolytic processing of the triggering receptor expressed on myeloid cells-2 (TREM2) protein by ectodomain shedding and γ-secretase-dependent intramembranous cleavage. J. Biol. Chem. *288*, 33027–33036.

Yeh, F.L., Wang, Y., Tom, I., Gonzalez, L.C., and Sheng, M. (2016). TREM2 binds to apolipoproteins, including APOE and CLU/APOJ, and thereby facilitates uptake of amyloid-beta by microglia. Neuron *91*, 328–340.

Yuan, P., Condello, C., Keene, C.D., Wang, Y., Bird, T.D., Paul, S.M., Luo, W., Colonna, M., Baddeley, D., and Grutzendler, J. (2016). TREM2 haplodeficiency in mice and humans impairs the microglia barrier function leading to decreased amyloid compaction and severe axonal dystrophy. Neuron *92*, 252–264.

Zhang, Y., Wang, N., Raab, R.W., McKown, R.L., Irwin, J.A., Kwon, I., van Kuppevelt, T.H., and Laurie, G.W. (2013). Targeting of heparanase-modified syndecan-1 by prosecretory mitogen lacritin requires conserved core GAGAL plus heparan and chondroitin sulfate as a novel hybrid binding site that enhances selectivity. J. Biol. Chem. *288*, 12090–12101.

Zhao, Y., Wu, X., Li, X., Jiang, L.L., Gui, X., Liu, Y., Sun, Y., Zhu, B., Piña-Crespo, J.C., Zhang, M., et al. (2018). TREM2 is a receptor for β-amyloid that mediates microglial function. Neuron *97*, 1023–1031.e7.

Zhong, L., Xu, Y., Zhuo, R., Wang, T., Wang, K., Huang, R., Wang, D., Gao, Y., Zhu, Y., Sheng, X., et al. (2019). Soluble TREM2 ameliorates pathological phenotypes by modulating microglial functions in an Alzheimer's disease model. Nat. Commun. *10*, 1–16.

Zimmermann, I., Egloff, P., Hutter, C.A.J., Kuhn, B.T., Bräuer, P., Newstead, S., Dawson, R.J.P., Geertsma, E.R., and Seeger, M.A. (2020). Generation of synthetic nanobodies against delicate proteins. Nat. Protoc. *15*, 1707–1741.

STAR★METHODS

KEY RESOURCES TABLE

REAGENT or RESOURCE	SOURCE	IDENTIFIER
**Antibodies**		
Rabbit monoclonal anti-TREM2 antibody	Abcam	Cat#ab209814
Biotinylated goat polyclonal anti-TREM2 antibody	R&D systems	Cat#BAF1828; RRID: AB_2208688
**Bacterial and Virus Strains**		
MACH1 T1	ThermoFisher	Cat#C862003
DH10Bac	ThermoFisher	Cat#10361012
**Chemicals, Peptides, and Recombinant Proteins**		
pTT5-SP-TREM2-6xC-His- (19-131)	This paper	N/A
pTT5-SP-TREM2-6xC-His-avi-(19-174)	This paper	N/A
pHTBV1.1-SecNH-Bio:TREM2-10xN-His-C-avi (19-174)	This paper	N/A
pFB-Sec-Bio5:TREM2 N6xHis-C-avi (19-131)	This paper	N/A
pFB-Sec-Bio5:TREM2 N6xHis-C-avi (19-138)	This paper	N/A
pFB-Sec-NH:TREM2 N-6xHis (19-174)	This paper	N/A
TREM2 standard peptide	SinoBiological	Cat#11084-H08H
TMB ELISA substrate	ThermoFisher	Cat# 34028
HRP conjugated streptavidin	ThermoFisher	Cat#N100
Alexa Fluor 647 NHS ester	ThermoFisher	Cat#A37573
**Deposited Data**		
scFv-4 with TREM2 (19-174)	This paper	PDB: 6Y6C
scFv-4 with TREM2 (19-131)	This paper	PDB: 6YMQ
scFv-2 with TREM2 (19-131)	This paper	PDB: 6YYE
SP140 PHD-Bromodomain complex with scFv (data phasing)	Fairhead et al., 2019	PDB: 6G8R
TREM2 Ig-domain (data phasing)	Sudom et al. (2018)	PDB: 5UD7
**Experimental Models: Cell Lines**		
HEK293	ATCC	Cat#CRL1573
HEK293 transfected with TREM2-DAP12	Thornton et al. (2017)	N/A
Expi293F (for protein expression)	ThermoFisher	Cat#A14527
Sf9 (for protein expression)	ThermoFisher	Cat#11496015
**Oligonucleotides**		
List of oligonucleotides used in this study	This study	Table S1
**Recombinant DNA**		
pHL-sec (Signal peptide origin)	Aricescu et al. (2006)	Addgene plasmid # 99845
pHTBV1.1-SecNH-Bio	Strain-Damerell et al. (2014)	N/A
pFB-Sec-Bio5	SGC	N/A
pFB-Sec-NH	SGC	Addgene plasmid # 39189
pTT5	Zhang et al. (2013)	Addgene plasmid # 52326
**Software and Algorithms**		
Phenix version 1.17.1	Emsley et al. (2010); Liebschner et al. (2019)	https://www.phenix-online.org/
ChimeraX version: 0.93	Goddard et al. (2018)	https://www.rbvi.ucsf.edu/chimerax/
Buster 2.10.3	Bricogne et al. (2016)	https://www.globalphasing.com/buster/
PyMOL 2.3.4	The PyMOL Molecular Graphics System, Version 2.3 Schrödinger, LLC	https://pymol.org/2/#download
GraphPad Prism version 8.42 for Windows	GraphPad Software, La Jolla California USA	https://www.graphpad.com

RESOURCE AVAILABILITY

Lead contact

Further information and requests for reagents and resources may be directed to and will be fulfilled by the Lead Contact Alex N. Bullock, (alex.bullock@cmd.ox.ac.uk)

Materials availability

Plasmids generated in this study will be deposited to Addgene and made available on request.

Data and code availability

The coordinates and structure factors for the crystal structures reported in this article have been deposited in the PDB with accession codes 6YYE (TREM2-scFv-2), 6Y6C (TREM2-scFv-4) and 6YMQ (TREM2-scFv-4).

EXPERIMENTAL MODEL AND SUBJECT DETAILS

Cell lines

All plasmids were generated in MACH1 or DH5α (Thermofisher Scientific C862003) cells grown in LB media at 37°C and bacmid DNA for insect cell expression and baculovirus generation were generated in DH10Bac (Thermofisher Scientific 10361012). For phage display, the enriched phages were amplified overnight by infection of *E. coli* XL1-blue (Stratagene, La Jolla, CA, USA).

Baculoviral protein expression was performed in Sf9 cells (Thermofisher Scientific 11496015) grown at 27°C in SF900 II-SFM media. Mammalian protein expression was performed in Expi293F^{TM} cells (female, Thermofisher Scientific A14527) in Expi293^{TM} expression medium (Thermofisher Scientific) at 37°C, 8% CO_2.

WT HEK293 (female; ATCC CRL1573) and HEK293 cells stably expressing WT hTREM2 and hDAP12 were cultured in DMEM (GIBCO) with 10% heat-inactivated foetal bovine serum (FBS) (Gibco) at 37°C, 5% CO_2. The cell culture medium for the HEK293 TREM2-DAP12 cells was supplemented with 1 mg/mL puromycin hydrochloride (Cayman Chemical).

METHOD DETAILS

Cloning of TREM2 and scFvs

Cloning of TREM2 for ELISA validation

DNA encoding human TREM2 (amino acids 19-131; gi|9507203; MGC) used for antigen generation and TREM2 (a.a. 19-138) for ELISA validation were cloned into the baculovirus secretion plasmid pFB-Sec-Bio5, which provides a C-terminal Avi-tag sequence for biotinylation. The stalk-containing TREM2 (a.a. 19-174) used for ELISA validation was cloned into baculovirus secretion plasmid pFB-Sec-NH which provides a N-terminal hexahistidine tag. See Table S1 for oligonucleotides and Key Resources Table for vectors and cell lines.

Cloning of TREM2 for SPR

A TREM2 construct containing the native signal peptide (a.a. 1-174) was cloned into the pTT5 vector, followed by an Avi tag and TEV-cleavable hexahistidine tag (GLNDIFEAQKIEWHEGSENLYFQSHHHHHH) at its C-terminus. The SP sequence was cloned from pHL-sec vector into pTT5 DNA inserts (Aricescu et al., 2006; Zhang et al., 2013).

Cloning of TREM2 for X-ray crystallography

For structural studies, human TREM2 (a.a. 19–131) was cloned into the mammalian expression vector pTT5, following an N-terminal signal peptide (MGILPSPGMPALLSLVSLLSVLLMGCVAETG) with hexahistidine tag preceeded by additional linker (a.a. GTK) at its C-terminus.

The longer construct TREM2 (a.a. 19-174) containing the stalk was inserted by ligation independent cloning (LIC) (Strain-Damerell et al., 2014) into the vector pHTBV1.1-SecNH-Bio, which provides an N-terminal Gp64 signal peptide and TEV-cleavable hexahistidine tag as well as a C-terminal Avi tag.

Cloning of scFvs for baculovirus expression

ScFv antibody fragments identified by phage display were cloned for baculovirus expression with scaffold specific primers into the vector pFB-Sec-NH.

Expression and purification of TREM2 and scFvs in baculovirus expression system

Bacmid generation and transfection

TREM2 viruses for baculovirus expression were generated by transfection with bacmid DNA:JetPrime (Polyplus) complexes. Sf9 cells at a density of $0.2x10^6$ in adherent culture were transfected for 4 hours (h) at 27°C followed by medium exchange to SF900 II-SFM containing 0.1% penicillin and streptomycin (Thermofisher Scientific) with 2% FBS (Thermofisher Scientific). P_0 viruses were harvested after 7 days at 27°C and 120 μL was used to infect 3 mL of Sf9 cells for P_1 virus amplification (72 h and 450 rpm shaking and throw 6 mm). Following another round of viral amplification, 0.75 L of Sf9 cells in SF900 II-SFM media were infected with virus P_2 at 7 mL/L at density $2x10^6$ cells/mL in glass flasks (27°C, 100 rpm for 72 h, throw 25 mm).

Purification of TREM2 for antigen generation and ELISA validation

The clarified supernatants were loaded onto 5 mL Ni-NTA (Qiagen)/per 1 L at 10 mL/min. Resin was washed with 20 column volumes (CV) of 50 mM HEPES pH 7.4 and 300 mM NaCl, 5% glycerol, 5 mM imidazole followed by 10 CV of 10 mM imidazole and 600 mM NaCl containing buffer. Proteins were eluted with buffer containing 500 mM imidazole. Proteins were purified further by size exclusion chromatography (Superdex 200,16/60) and *in vitro* biotinylated using recombinant BirA enzyme (enzyme to substrate ratio of 1:50, w/w). The reaction was performed in a buffer containing 10 mM Tris-HCl, 50 mM NaCl and 10 mM MgOAc, pH 8.0 for 14 h, at 4°C in the presence of 0.1 mM biotin and 10 mM ATP. Subsequently, the buffer was exchanged by dialysis to 20 mM HEPES, 300 mM NaCl, 5% glycerol and 0.5 mM TCEP, pH 7.5

TREM2 (His19-Ala138) and (His19-Ser174) for ELISA validation were purified in the same manner but were stored at -80°C after size exclusion.

Expression and purification of scFvs

Viruses for scFvs were generated as above. Immobilised metal affinity chromatography (IMAC) was performed as for TREM2, except for a change to pH 6.8 which decreased precipitation. Eluted proteins were cleaved with TEV protease overnight (O/N) at 4°C followed by loading onto a 1 mL Protein A FF (GE healthcare) column at 3 mL/min. Resin was washed with 20 CV of base buffer (BB: 25 mM HEPES pH 7.5, 150 mM NaCl) followed by a wash with 10 CV of BB containing 0.05% Triton-X. Proteins were eluted with 0.1 M acetic acid pH 3 at 0.5 mL/min and immediately buffer exchanged on PD10 columns (scFv-1: 120 mM Citrate pH 7 with 50 mM L-arginine,50 mM L-glutamic acid, scFv-2: 100 mM HEPES pH 7.2, 25 mM NaCl, 100 mM L-arginine, 100 mM L-glutamic acid, scFv-3: 100 mM Tricine pH 8, scFv-4: 100 mM Tricine pH 8) and frozen until further use. Later, scFv-3 and scFv-4 oligomers were separated by double SEC on Superdex 200 16/60 in 20 mM Tris-HCl pH 7.5, 300 mM NaCl and 5% glycerol. Separated oligomers were pooled and buffer exchanged to PBS using vivaspin 2 10 MWCO concentrators (GE healthcare). Proteins were clarified by centrifugation at 30,000 x *g* and immediately frozen in liquid nitrogen and used for the assay within a week.

Expression and purification of TREM2 in mammalian cells

Transient transfection of TREM2 plasmids

For crystallisation studies of TREM2 (a.a. 1-131), Expi293F^{TM} cells (Thermofisher Scientific A14527) in Expi293^{TM} expression medium (Thermofisher Scientific) at 2.5x10^{6} were transfected with a mixture of 1 mg of pTT5:TREM2 (19-131) DNA to 6 mg L-PEI 25 K (Polysciences) per 1 L of culture. 50 mL of DNA and 50 mL of L-PEI in Opti-MEM (Thermofisher Scientific) was mixed and incubated for 15 min before addition to the culture. 1 mg/L kifunensine (Carbosynth) was added followed by 1 mM valproic acid (VPA), 20 h post-transfection. Cells were grown in vented flasks at 100 rpm (throw 25 mm), 37°C, 8% CO_2 for 72 h.

Purification of TREM2 for crystallisation

Culture supernatants were collected and TREM2 (a.a 19-131) was purified by affinity chromatography (Ni-NTA, Qiagen) in buffers containing 20 mM Tris-HCl pH 8, 500 mM NaCl, 5% glycerol followed by size exclusion chromatography (Superdex 75 10/30GL, GE Healthcare). The purified protein was deglycosylated with EndoH (PE019, Novoprotein) in 50 mM sodium citrate pH 5.5 overnight at 4°C, at the ratio of 1 mg protein: 2000 U EndoH. EndoH was removed using a second Ni-NTA affinity column, and the purified protein was stored in buffer containing 50 mM Tris pH 8.5, 200 mM NaCl, and 5% glycerol at 10 mg/mL concentration.

The longer TREM2 (a.a. 19-174) for crystallisation was expressed and purified similarly but transfection was performed with DNA to L-PEI 1:3 ratio and cells were grown in 2 L roller bottles (Greiner Bio-one). Affinity chromatography was performed in base buffer: 20 mM HEPES 7.4, 300 mM NaCl, 5% glycerol and proteins were immediately deglycosylated in solution adjusted with sodium citrate (final buffer pH 6). Size exclusion was performed in 20 mM HEPES 7.4, 150 mM NaCl, 5% glycerol on Superdex 200 16/60 (GE Healthcare).

Purification of TREM2 for SPR

Fully glycosylated biotinylated pTT5-TREM2 (19-174) protein for SPR studies was purified as described for TREM2 (a.a. 19-131) without the deglycosylation step. The Ni-NTA purified protein was biotinylated overnight at 4°C in the presence of 0.5 mM biotin, BirA (1:50), 1 mM ATP, and 7.5 mM $MgCl_2$. TREM2 was further purified by a second Ni-NTA affinity column and stored in buffer containing 50 mM Tris pH 8.5, 200 mM NaCl, and 5% glycerol.

Antibody selection and validation

Antibody generation through phage display selection was performed as described previously, using the *in vitro* biotinylated TREM2A (19-131) from Sf9 cells as antigen and a human synthetic single-chain fragment variable (scFv) library (Preger et al., 2020). Briefly, four rounds of selections were performed using streptavidin beads (Dynabeads M-280 streptavidin, Invitrogen) to immobilise the biotinylated TREM2 antigen. In the first two rounds, phages were incubated with immobilized antigen after a pre-selection on naked beads. In round 3 and 4, phage and antigen were incubated in solution before capturing on beads. The antigen-phage incubation time was decreased from 3 h in the first round to 1.5 h in rounds 2, 3, and 4. Furthermore, the selection pressure was augmented by increasing the number of washing steps and decreasing the amount of antigen added (200, 100, 50 and 10 pmol, respectively) between the different rounds. The enriched phages were recovered using trypsin digestion and amplified overnight by infection of *E. coli* XL1-blue. Amplification in round 1 was done using agar plates and in rounds 2, 3 and 4 in solution. Amplified phages were precipitated using PEG/NaCl and used for the next round of selection. Phagemid DNA from rounds 3 and 4 were purified and the scFv genes were transferred to an expression vector as described previously (Preger et al., 2020).

Following selections, cloning and transformation, a total of 188 colonies were picked from rounds 3 and 4 and analysed further. ELISA revealed 8 colonies as potential binders, of which 5 were identified as unique clones after DNA sequencing. These five were considered suitable for further validation, which was performed as previously described including ELISA, HTRF, Luminex, and SPR (Preger et al., 2020). Based on these results (Figure S1, HTRF data not shown), four scFv candidates, scFv-1, scFv-2, scFv-3, scFv-4, were selected to be produced in large-scale and tested in additional binding kinetics and co-crystallisation trials.

ScFv binding kinetics

ScFv-1 was determined to be a weaker binder of TREM2 (Figure S1) and thus was not analysed in detail. Approximate binding kinetics of Protein A purified ScFv-2, scFv-3 and scFv-4 were determined by SPR performed on a Biacore 8K instrument. Biotinylated TREM2 (19-174) produced in Expi293F^{TM} human cells was immobilised on a streptavidin-coated chip supplied in the Biotin CAPture kit (28-9202-34, GE Healthcare) at 0.5 μg/mL concentration. ScFvs were diluted in 10 mM HEPES, 150 mM NaCl, 0.05% P-20 and injected at a flow rate of 30 μL/min for 60 seconds followed by dissociation for 120 seconds. The binding response was calculated after subtracting signal coming from a blank flow cell and the buffer. Langmuirian 1:1 model was used to calculate the binding kinetics. For scFv-3 and scFv-4 avidity contributed to obtained values. Additionally, scFv-4 showed some non-specific interaction with the reference sensor, and so calculated values are used as approximation only.

Crystallisation

scFv-4 PDB: 6YMQ

After Protein A purification, scFv-4 in 100 mM Tricine, pH 8 and TREM2 (a.a. 1-131) were mixed at molar ratio 1:1.1 and buffer exchanged into 20 mM Tricine pH 8, 200 mM NaCl, 200 mM L-arginine, 200 mM L-glutamic acid. The complex was concentrated to 14 mg/mL and crystals were grown by sitting drop vapour diffusion in solution containing 39% MPD, 0.2 M ammonium acetate, 0.1 M citrate pH 5.5 at 20°C. Crystals were mounted directly from the drop and vitrified in liquid nitrogen.

scFv-4 PDB: 6Y6C

Antibody scFv-4 was buffer exchanged into 25 mM HEPES, 300 mM NaCl and 5% glycerol and mixed with TREM2 (a.a. 1-174) at molar ratio 1:1.1. The complex was concentrated to 12.5 mg/mL and crystal plates were set up after 32,000 x *g* spin for 20 min. Protein crystals appeared after 1 day using a reservoir solution containing 0.2 M potassium chloride, 35% pentaerythritol propoxylate 5/4, 0.1 M HEPES pH 7.5 at 20°C. Crystals were cryo-protected after addition of reservoir solution containing 20% ethylene glycol and vitrified in liquid nitrogen.

scFv-2 PDB: 6YYE

Protein A purified antibody scFv-2 in 100 mM citrate buffer and 250 mM L-arginine and 50 mM L-glutamic acid was mixed with TREM2 (a.a 19-131) at a molar ratio 1.3:1 and buffer exchanged on PD10 column to 60 mM Tricine 8, 200 mM NaCl containing 200 mM L-arginine, 200 mM L-glutamic acid. The complex was concentrated to 16 mg/mL and precipitation was removed by centrifugation. Crystals were grown in 15 mM nickel chloride, 0.1 M Tris pH 8.5, 2.2 M ammonium acetate at 20°C. Crystals were cryo-protected with reservoir containing 20% ethylene glycol before vitrification.

Data collection and structure determination

For scFv-4 structures (6YMQ, 6Y6C), data were collected on beamline I24 at the Diamond Light Source using the X-ray wavelength of 0.9688Å and processed using Xia2 package and DIALS (G. Winter 2010; Winter et al., 2018).

For the lower resolution scFv-2 dataset (6YYE), data were collected on beamline I03 at the Diamond Light Source using the X-ray wavelength 0.9763 Å. The initial model was built using PhenixRefine and coot modelling using Xia and DIALS dataset. Later the model was used to refine against AutoProc processed dataset with ellipsoidal truncation by Staraniso (Evans, 2006; Kabsch et al., 2010; Vonrhein et al., 2011, 2018). The model was finished by refinement in Buster v.2.10.3 (Bricogne et al., 2016) and final rounds of Phenix refine (Liebschner et al., 2019).

The first dataset of scFv-2 (6YYE) was phased using the structure of another scFv (PDB: 6G8R) generated from the same library and solved previously (Fairhead et al., 2019). TREM2 was phased using PDB 5UD7 (Sudom et al., 2018). The resulting scFv-2 co-structure (PDB 6YYE) was used for phasing of PDB 6YMQ. Phenix molecular replacement was used to phase the data and coot to build a model (Emsley et al., 2010; Liebschner et al., 2019). Models were improved by iterative cycles of Phenix Refine and coot rebuilding.

	6Y6C (scFv-4)	6YMQ (scFv-4)	6YYE (scFv-2)
Ramachandran statistics			
Ramachandran favoured (%)	97.21	92.59	93.73
Ramachandran allowed (%)	2.79	7.41	5.97
Ramachandran outliers (%)	0.0	0.0	0.3
Rotamer outliers (%)	0.35	0.92	2.35
Clash score	2.56	4.76	6.01

Molecular graphics and analyses were performed with UCSF ChimeraX, developed by the Resource for Biocomputing, Visualization, and Informatics at the University of California, San Francisco, with support from National Institutes of Health R01-GM129325 and the Office of Cyber Infrastructure and Computational Biology, National Institute of Allergy and Infectious Diseases (Goddard et al., 2018). Crystal lattice images were generated using PyMOL (Schrödinger, LLC.). See Key Resources Table for all software.

Mammalian cell culture for cell biology

The HEK293 TREM2-DAP12 cell line was a kind gift from Professor Peter St-George Hyslop and generated as described previously (Thornton et al., 2017). HEK293 wild type cell line was purchased from ATCC (CRL1573).

WT HEK293 and HEK293 cells stably expressing WT hTREM2 and hDAP12 were cultured in T-175 flasks (Greiner Bio-one) in DMEM (GIBCO) with 10% heat-inactivated foetal bovine serum (FBS) (Gibco) at 37°C, 5% CO_2. The cell culture medium for the HEK293 TREM2-DAP12 cells was supplemented with 1 mg/mL puromycin hydrochloride (Cayman Chemical).

sTREM2 ELISA

HEK293 transfected with TREM2-DAP12 were plated in 96-well plates (Corning Biocoat, 354640) at $30x10^3$ cells per well, grown for 24 h, washed with culture medium and treated with antibodies for 5 h. After antibody incubation, the medium was collected and transferred into a new plate, centrifuged at 1,000 x *g* followed by supernatant transfer into a fresh plate that was stored at -80°C.

TREM2 ELISA was performed by coating high bind 96-well plates (Sigma, M4436-040EA) with 8 μg/mL TREM2 antibody (Abcam, ab209814) O/N at 4°C followed by 1 h blocking using PBS with 1% BSA (Sigma). 100 μL of the defrosted cell supernatants were added at the appropriate dilution in DMEM + 1% BSA alongside a standard curve with TREM2 peptide (SinoBiological, 11084-H08H) and incubated for 2 h, at RT. 25 μL of the biotinylated detection antibody (R&D, BAF1828) at 1.5 μg/mL was added for an hour followed by the addition of HRP conjugated streptavidin diluted at 1:30,000 (ThermoFisher Scientific, N100). TMB ELISA substrate (ThermoFisher Scientific 34028) was added for 20 min followed by 2 M sulphuric acid (Immuno Chemistry technologies). Results were obtained by measuring optical density (OD) at 450 nm using a plate reader (SpectraMax M2). OD measurements in the linear range (0.8-1.9) were chosen for analysis and TREM2 concentration was interpolated from the TREM2 standard curve. To ensure that scFv binding to TREM2 did not interfere with the assay antibodies, the standard peptide (a.a 1-174, SinoBiological 11084-H08H) was mixed with each one of the scFvs in DMEM +1% BSA and results were compared to the standard curve only. Also, as another control, to assess TREM2 epitope recognition by the ELISA antibodies, TREM2 proteins (His19-Ala138 and His19-Ser174) were diluted to 2441 pg/mL in DMEM +1% BSA using five-point serial dilutions and analysed using the protocol described above. Additionally, the medium conditioned in cells for 5 h was spiked with scFvs and ELISA was performed in comparison to non-spiked sample (data not shown).

Alexa fluor 647 cell staining and imaging

Purified scFv-3 and scFv-4 oligomers and control non-TREM2 scFvs were incubated with Protein A and Alexa Fluor 647 NHS ester (Thermofisher Scientific A37573) at 1:10 molar ratio for 20 h, at 4°C and excess dye was removed by NAP5 columns (GE healthcare). HEK293 and HEK293 transfected with TREM2-DAP12 were prepared as for the ELISA and treated with scFv-4 for 5 h. Cells were fixed in 4% paraformaldehyde in PBS (Santa Cruz Biotechnology) for 15 min and kept at 4°C until imaging. Nuclei were stained with Hoechst 33342 for 1 h in the dark and cells were imaged using an OperaPhenix confocal microscope with 40 x magnification, 405/456 nm and 640/706 nm, excitation/emission respectively.

QUANTIFICATION AND STATISTICAL ANALYSIS

All statistical analyses were carried out using three independent biological replicates and two or three technical repeats unless specifically stated otherwise. Error bars are standard deviation (SD) and EC_{50} was interpolated using non-linear regression (inhibitor vs. response- four parameters) in GraphPad Prism Software 8.4.2. Each statistical test was performed on raw data normalised to cell number or signal before normalisation to the percentage of control was performed. One-way ANOVA with Dunnett's test comparison to control untreated cells was utilised to calculate significance. p values are indicated in the respective figure legends with symbols (*P <.05 and **P <.01; ****P <.0001).

Structure, Volume *29*

Supplemental Information

Selection and structural characterization of anti-TREM2 scFvs that reduce levels of shed ectodomain

Aleksandra Szykowska, Yu Chen, Thomas B. Smith, Charlotta Preger, Jingjing Yang, Dongming Qian, Shubhashish M. Mukhopadhyay, Edvard Wigren, Stephen J. Neame, Susanne Gräslund, Helena Persson, Peter J. Atkinson, Elena Di Daniel, Emma Mead, John Wang, John B. Davis, Nicola A. Burgess-Brown, and Alex N. Bullock

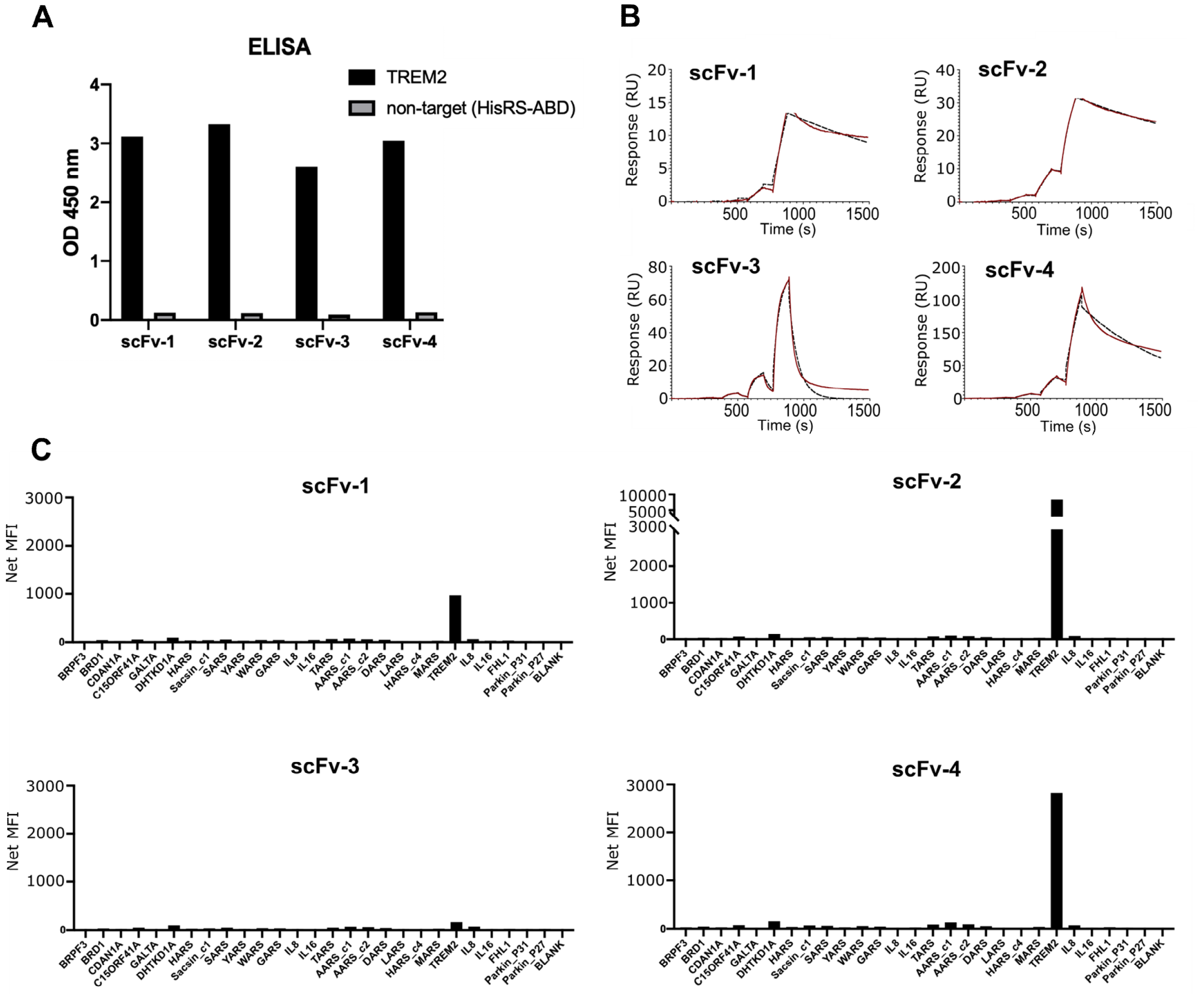

Figure S1. Initial validation of scFvs against TREM2, Related to STAR Methods. (A) Validation of scFvs binding to TREM2 by ELISA. Antigen TREM2 (a.a. 19-131) purified from insect cells (black bars) or a non-target protein (HisRS-ABD, grey bars) was added to a streptavidin-coated plate, followed by the addition of scFvs (in bacteria supernatant). Bound scFv were detected using anti-FLAG M2 HRP-conjugated antibody and TMB substrate. (**B**) Initial SPR Single Cycle Kinetic measurements of scFvs purified from *E. coli* using Biacore T200 (Cytiva). An anti-FLAG M2 antibody was immobilised on a CM5 sensor chip and FLAG-tagged scFv was captured on the surface. The same TREM2 antigen was injected with increasing concentrations (0.16, 0.8, 4, 20 and 100 nM*).* Response was measured in resonance units (RU) over time (s). Red lines represent the measured data values, and dashed black lines represent the fit of the curve based on a 1:1 Langmuir binding model. (**C**) Luminex binding of scFvs against a panel of 27 proteins including TREM2. Proteins were added to neutravidin coupled magnetic colour-coded beads before addition of scFv (bacteria supernatant). Binding was detected using an RPE-conjugated anti-FLAG M2 antibody and analysed using FLEXMAP 3D (Luminex corp.) recording median fluorescence intensity (MFI).

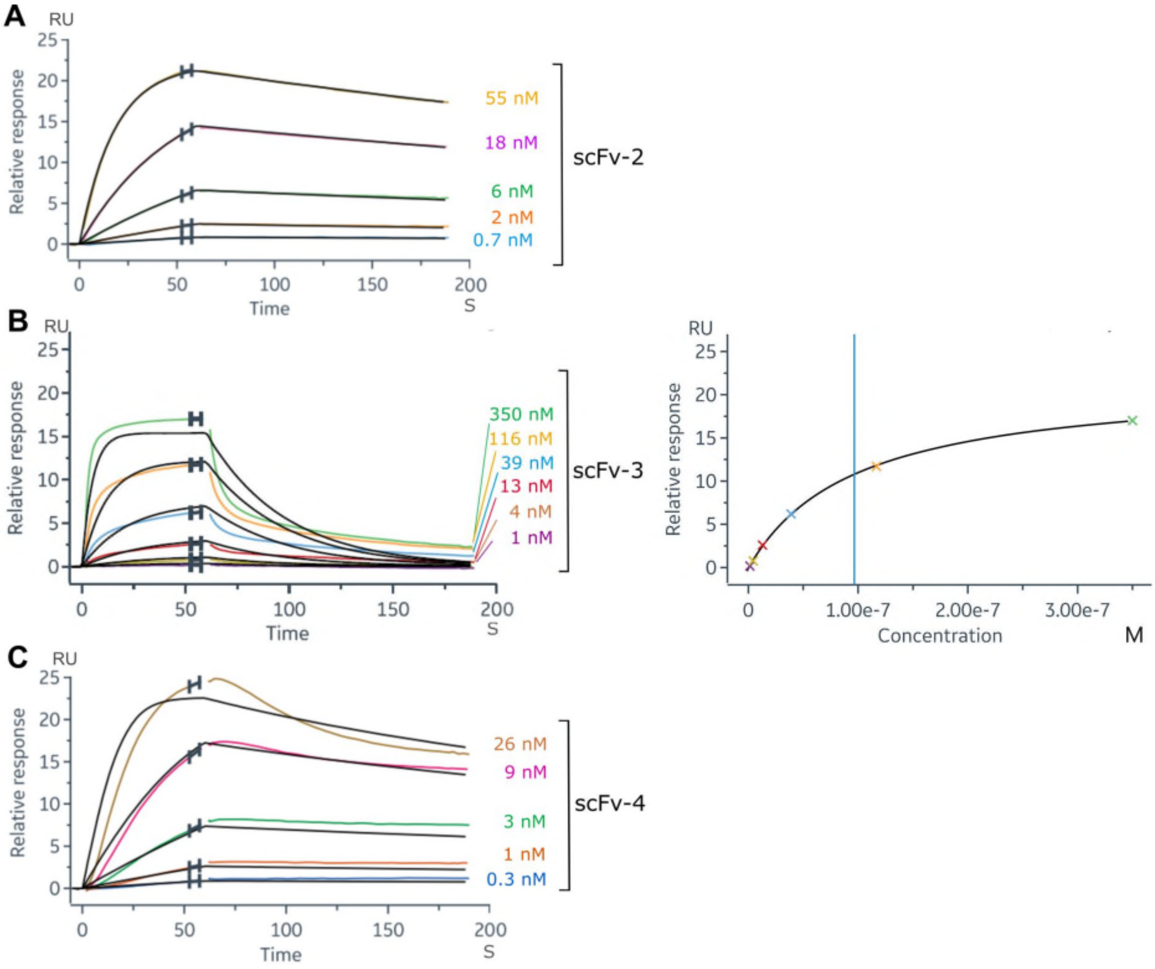

Figure S2. Kinetic measurements of three anti-TREM2 scFvs and estimation of apparent K_D values, Related to STAR Methods. Binding kinetics were determined by surface plasmon resonance (SPR) performed on a Biacore 8K instrument. ScFvs were produced in insect cells and showed a mix of monomeric and dimeric species. Biotinylated TREM2 (His19-Ser174) produced in Expi293F™ (Thermofisher Scientific, A14527) was immobilised using the Biotin CAPture Kit (Cytiva) and the binding of different concentrations of scFv was analysed. The binding kinetics of (**A**) scFv-2, (**B**) scFv-3 and (**C**) scFv-4 to TREM2 were fitted using a Langmuirian 1:1 model. ScFv-2: k_{on} = 9.9 x 10^5 $M^{-1}s^{-1}$, k_{off} = 1.6 x 10^{-3} s^{-1}, ${}^{App}K_D$ = 1.6 nM; ScFv-3: k_{on} = 5.0 x 10^5 $M^{-1}s^{-1}$, k_{off} = 2.7 x 10^{-2} s^{-1}, ${}^{App}K_D$ = 54 nM; ScFv-4: k_{on} = 5.0 x 10^6 $M^{-1}s^{-1}$, k_{off} = 2.9 x 10^{-3} s^{-1}, ${}^{App}K_D$ = 0.6 nM. The data for scFv-3 fit relatively poorly to a 1:1 model. However, steady state equilibrium analysis (right panel) yielded similar ${}^{App}K_D$ = 96 nM. Some non-specific interaction to the reference chip was observed for both scFv-3 and scFv-4. The k_{on} and k_{off} may also contain an avidity component, especially in the case of scFv-3 and scFv-4, so ${}^{App}K_D$ is used to describe the results. Association and dissociation curves are coloured for each concentration and the fitted curves are drawn in black. Competitive binding studies suggested that scFv-3 and scFv-4 compete for binding to TREM2 (data not shown).

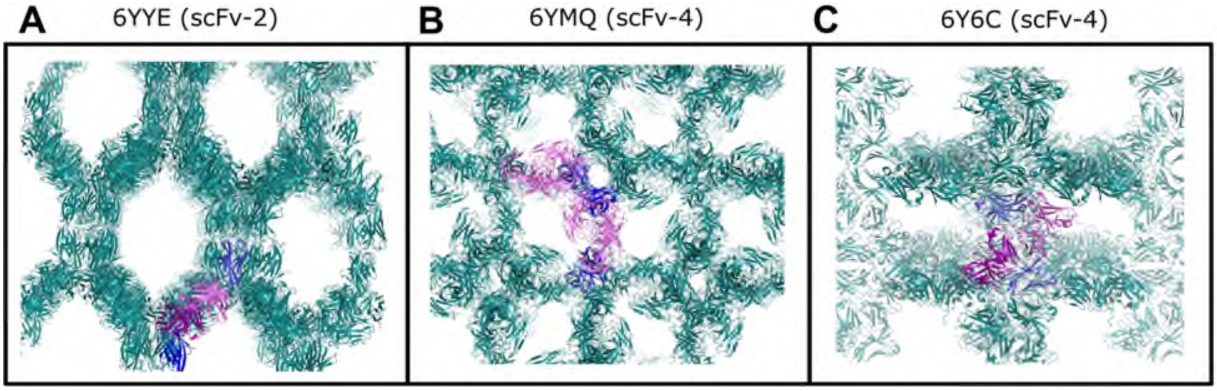

Figure S3. The crystal lattices of scFvs and TREM2 complexes, Related to Figure 3. (**A**) Crystal lattice of scFv-2 and TREM2 complex (6YYE) crystallised in unit cell of 114, 126, 225 Å with 77% solvent composition. Lattice was visualised by generating symmetry mates of components of an asymmetric unit consisting of 2 subunits of scFv (pink) and six subunits of TREM2 (blue). (**B**) Crystal lattice of the scFv-4 and TREM2 complex (6YMQ) crystallised in unit cell of 167, 181, 125 Å with 69% solvent composition. Green lattice visualised by generating symmetry mates of components of an asymmetric unit consisting of 6 subunits of scFv (pink) and six subunits of TREM2 (blue) (**C**) Crystal lattice of scFv-4 and TREM2 complex (6Y6C) crystallised in unit cell of 112, 112, 232 Å with 74% solvent composition. Lattice was visualised by generating symmetry mates of components of an asymmetric unit consisting of 2 subunits of scFv (pink) and two subunits of TREM2 (blue).

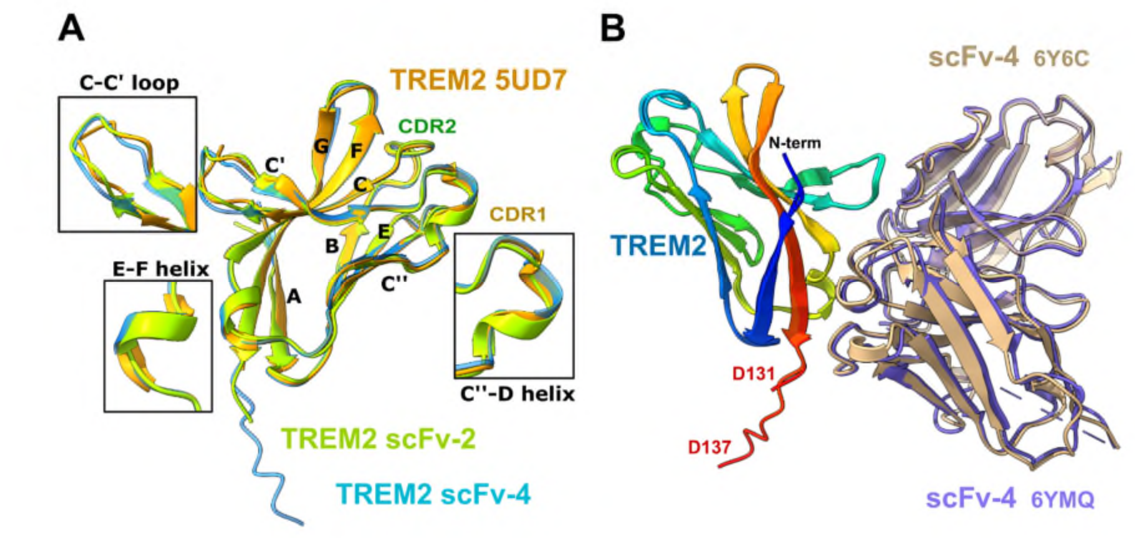

Figure S4. Superimposition of selected TREM2 and scFv-2 and scFv-4 co-structures, Related to Figure 3. **(A)** Superimposition of the highest resolution structure of TREM2 (2.2 Å) previously deposited in the PDB, 5UD7 (Sudom *et al.*, 2018), and TREM2 bound to scFv-2 and scFv-4. The root mean square deviation (RMSD) of C-alpha atoms comparing 5UD7 to the TREM2 complexes with scFv-2 and scFv-4 is 0.7 Å and 0.4 Å, respectively. The loops displaying the highest variation are shown in inset panels and named by secondary structure or TREM2 CDR. Amongst them is the C-C' loop which is differentially bound by the scFvs as well as the CDR2 loop which shows a variable extent of a short α-helix, especially when bound to scFv-2. TREM2 5UD7 is coloured in orange, scFv-4-bound TREM2 in blue and scFv-2-bound TREM2 in green. **(B)** Superposition of the two TREM2-scFv-4 structures showed excellent agreement (Cα RMSD = 0.5 Å). ScFv-4 in complex with TREM2 His19-Ser174 (6Y6C) is shown in tan, whereas scFv-4 in complex with TREM2 His19-Asp131 (6YQM) is shown in purple.

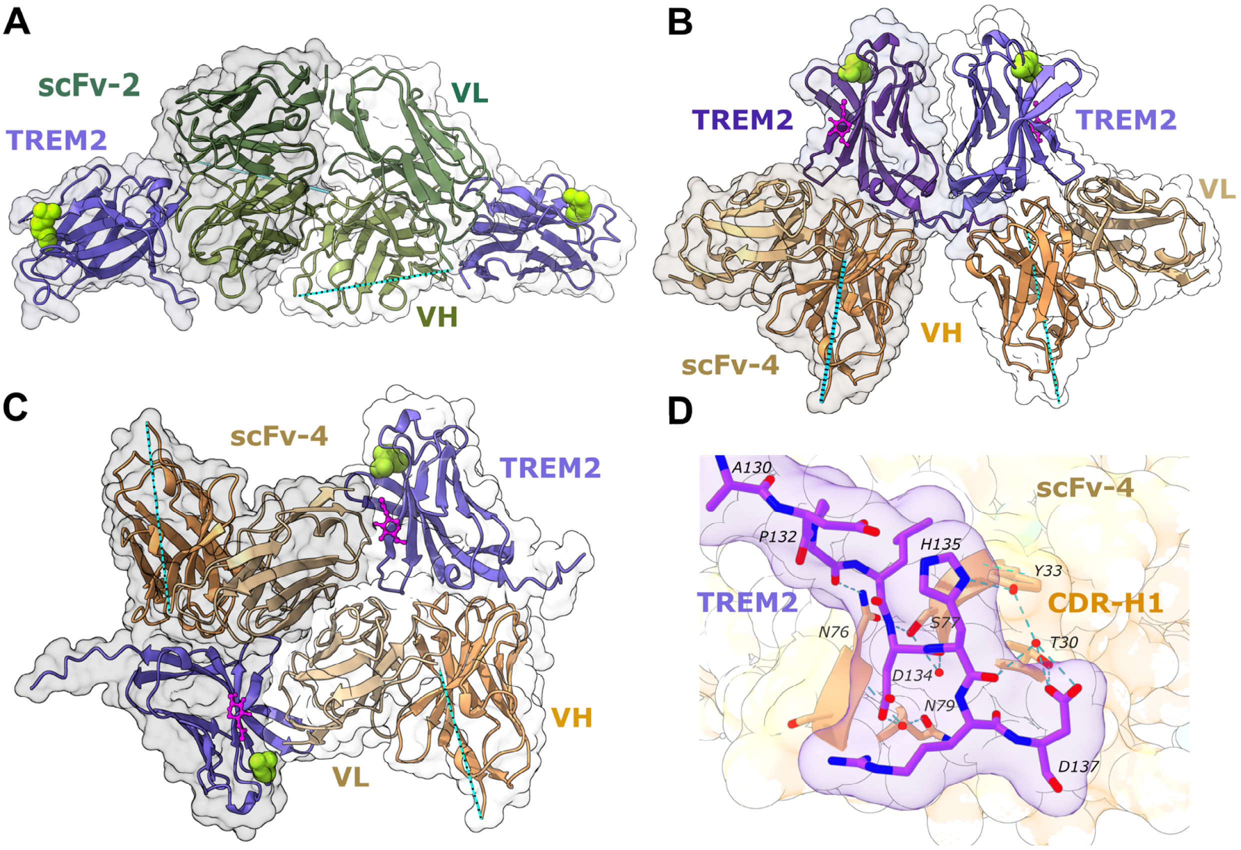

Figure S5. Different crystal packing of TREM2-scFv2 and scFv-4 complexes, Related to Figure 3. (**A**) Crystal packing between scFv-2 subunits in the TREM2 complex (PDB 6YYE). The scFv-2 VH and VL domains are coloured in different shades of green; The disordered linker between VH and VL domains is shown by a dotted cyan line. TREM2 is coloured slate blue. A molecular surface representation of each TREM2-scFv-2 complex is shown in a different transparent shade of grey. The TREM2 side chain Arg47 is shown in green spacefill for reference. (**B**) Crystal packing between TREM2 (a.a. 19-174, slate blue) and scFv-4 subunits (tan)(PDB 6Y6C). Each TREM2 subunit interacts with two scFv-4 molecules through interactions from the TREM2 ectodomain and stalk region, respectively. N-acetylglucosamine (NAG) is coloured in pink and presented as ball and stick (**C**) Crystal packing between scFv-4 subunits (tan) observed in the TREM2 (a.a. 19-174) co-structure (PDB 6Y6C). Each scFv-4 subunit packs against two molecules of TREM2 by interaction with the primary TREM2 epitope as well as an additional surface consisting of the TREM2 C-C' and F-G loops. (**D**) The TREM2 C-terminal stalk sequence Pro132-Asp137 (purple) packs against ScFv-4 CDR-H1 and Asn76-Asn79 (tan). Residues forming hydrogen bond interactions are presented as sticks and waters as red spheres. Hydrogen bonds are shown as blue dashed lines.

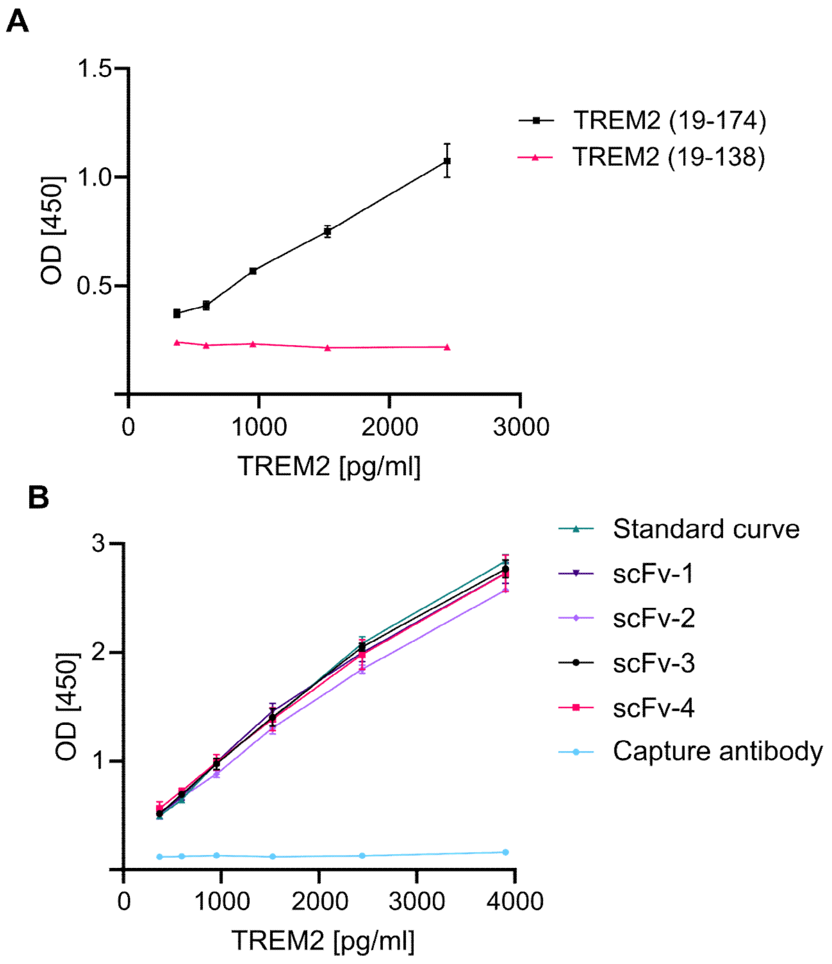

Figure S6. Validation of the sTREM2 ELISA assay, Related to Figure 6. (**A**) Identification of the soluble TREM2 amino acid sequence essential for ELISA detection. ELISA was performed using TREM2 (a.a. 19-174) protein containing the immunoglobulin domain and stalk and a shorter Ig domain only TREM2 protein (a.a. 19-138) in order to determine the requirement for the stalk sequence. The data represent n=1, 2 technical repeats. (**B**) Characterisation of epitope compatibility between ELISA antibodies and scFvs. Each of the four scFvs was incubated at 10 µg/mL with 3906 pg/mL standard curve peptide for 15 min. Dilution series were used to generate curves and a standard curve containing TREM2 peptide only was used for comparison. The capture antibody added to the standard curve is used to demonstrate the effects of overlapping epitopes. The data represent n=1, 2 technical repeats, except for scFv-3 and scFv-4 where n=2 independent experiments.

Table S1. Oligonucleotide sequences used for cloning, related to Key Resources Table

OLIGONUCLEOTIDE	SOURCE	IDENTIFIER
scFvs scaffold-fwd	SourceBioscience	TACTTCCAATCCATGGAGGTGCAATTGTTGGAGAGC
scFvs scaffold-rvs	SourceBioscience	TATCCACCTTTACTGTCATTTGATCTCCAGCTTGGT
pHL-sec-signal peptide forward	SourceBioscience	ATGGGGATCCTTCCCAGCCCTGGGATGCCTGCGCTGCTCTCCCTCGTGAGCCTTCTCT
pHL-sec-signal peptide reverse	SourceBioscience	ACCGGTTTCAGCTACGCAACCCATCAGCAGCACGGAGAGAAGGCTCACGAGGGAGAG
pTT5-TREM2 (19-131) forward	SourceBioscience	GATGGGTTGCGTAGCTGAAACCGGTCACAACACCACCGTGTTCCAGGGCGT
pTT5-TREM2 (19-131) reverse	SourceBioscience	CTCGAGTGATCATTAGTGATGGTGATGGTGGTGCTTGGTACCGTCTGCCAGCACCTCCACCAGGAC
pTT5-TREM2 (1-174) forward	SourceBioscience	ACGGATCTCTAGCGAATTCACCATGGAGCCTCTGCGCCTGCTGATC
pTT5-TREM2(1-174) reverse	SourceBioscience	CAATTTTCTGAGCTTCGAAGATGTCGTTCAGACCGGAAGTCGGTGGGAACGGGATTTCAC
pHTBV1.1sec-TREM2 (19-174) reverse	SourceBioscience	TATCCACCTTTACTGCTGGAAGTGGGTGGGAAGGG
pHTBV1.1sec-TREM2 (19-174) forward	SourceBioscience	TACTTCCAATCCATGCACAACACCACAGTGTTCC
pFB-sec-bio-TREM2 (1-131) forward	SourceBioscience	As above
pFB-sec-bio-TREM2 (1-138) forward	SourceBioscience	As above
pFB-sec-bio-TREM2 (1-131) reverse	SourceBioscience	TATCCACCTTTACTGCTGTCTGCCAGCACCTCCAC
pFB-sec-bio-TREM2 (1-138) reverse	SourceBioscience	TATCCACCTTTACTGCTAGCATCCCGGTGATCCAG
pFB-sec-NH-TREM2 (1-174) forward	SourceBioscience	TTAAGAAGGAGATATACTATGGAGCCTCTCCGGCTGCTC
pFB-sec-NH-TREM2 (1-174) reverse	SourceBioscience	GATTGGAAGTAGAGGTTCTCTGCCCCGGGGAACCAGAGATC
